# Supplementary figures and images for: Negation mitigates rather than inverts the neural representations of adjectives
Source: PLoS Biol. 2024 May 30;22(5):e3002622. doi: 10.1371/journal.pbio.3002622 (PMC11139306; doi:10.1371/journal.pbio.3002622)

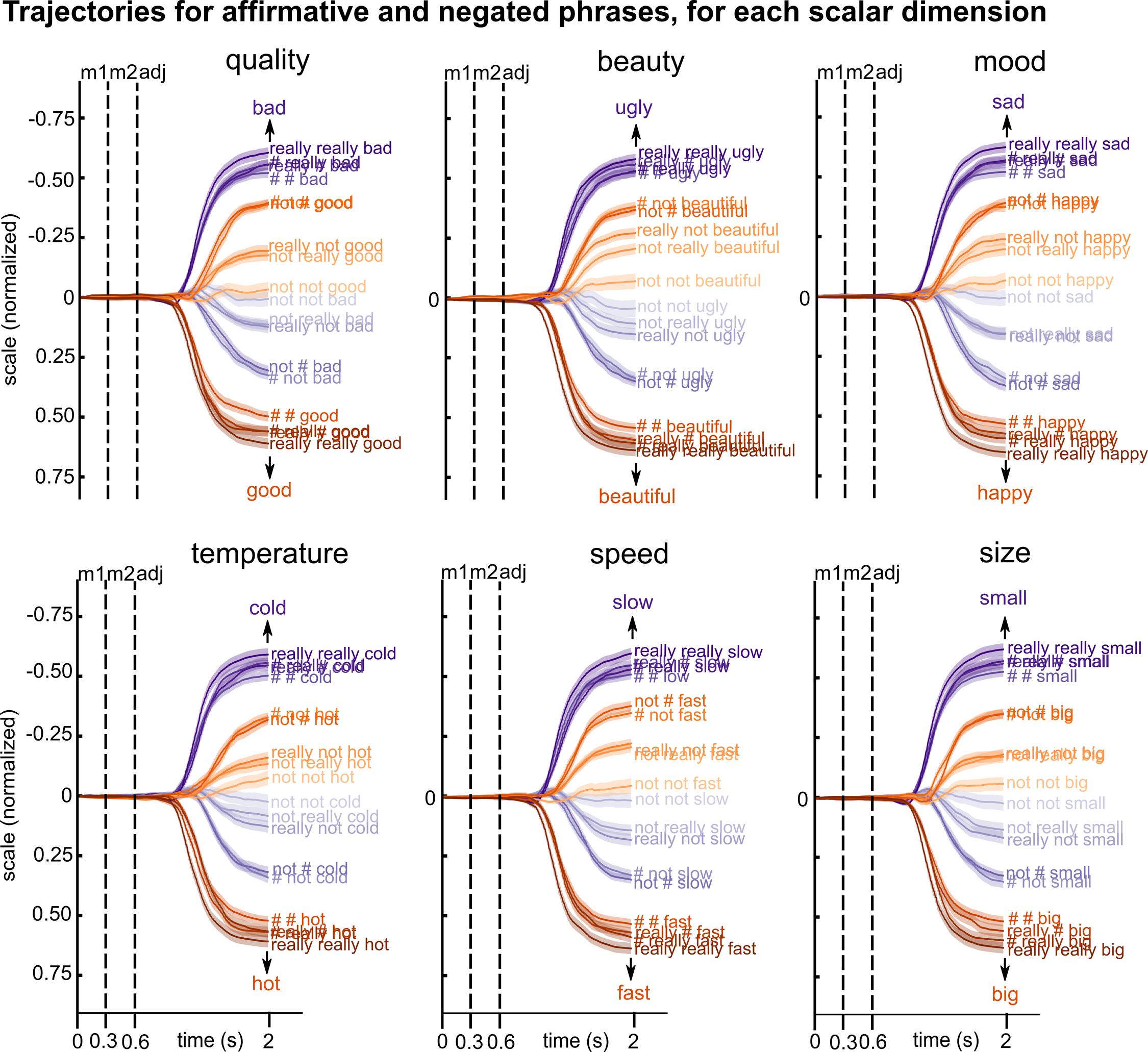

Supplement: S1 Fig — Behavioral trajectories for low (purples) and high (oranges) antonyms over time, for each scalar dimension (i.e., quality, beauty, mood, temperature, speed, and size), for each modifier (shades of orange and purple), and for affirmative and negated phrases. Black vertical dashed lines indicate the presentation onset of each word: modifier1, modifier2, and adjective. (TIF) [file pbio.3002622.s001.tif]

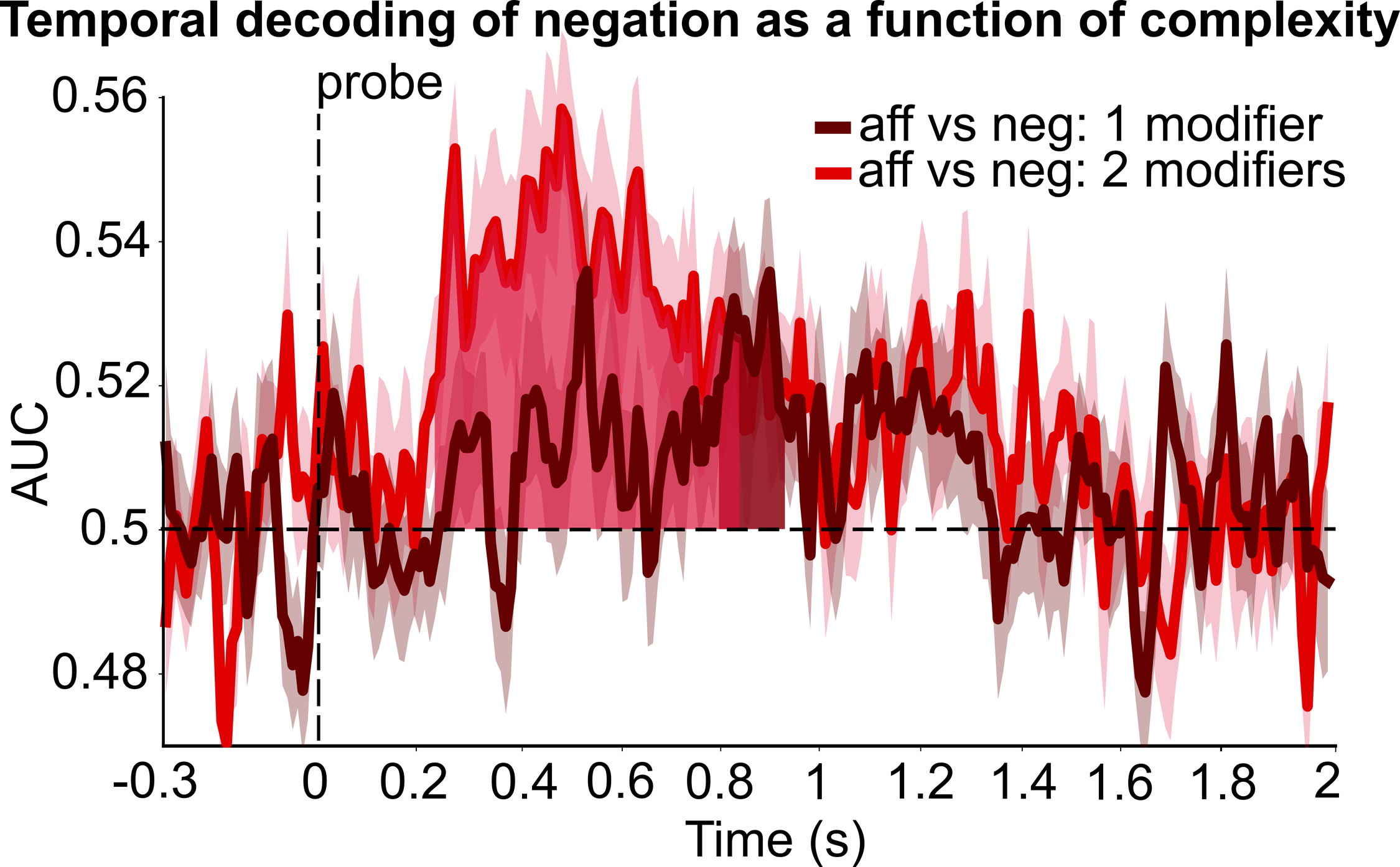

Supplement: S2 Fig — Decoding accuracy of negation over time, as a function of the number of modifiers (1 modifier: dark red line and shading; 2 modifiers: light red line and shading). Significant time windows are indicated by dark red (1 modifier) and light red (2 modifiers) shading. These results show that we could significantly decode the difference between affirmative and negated phrases between 230 and 930 ms after the onset of the probe, especially when the phrase included two modifiers (1 modifier: between 790 and 930 ms: p < 0.001; 2 modifiers: between 230 and 840 ms: p < 0.001). This suggests that the representation of modifiers is reactivated at the stage when participants have to perform the yes/no task. 1 modifier: “really ###,” “### really,” “not ###,” “### not”; 2 modifiers: “really really,” “really not,” “not really,” “not not.” AUC = area under the receiver operating characteristic curve, chance = 0.5 (black dashed horizontal line); the black vertical dashed line indicates the presentation onset of the probe; aff = affirmative, neg = negated; each line and shading represent participants mean ± SEM. (TIF) [file pbio.3002622.s002.tif]

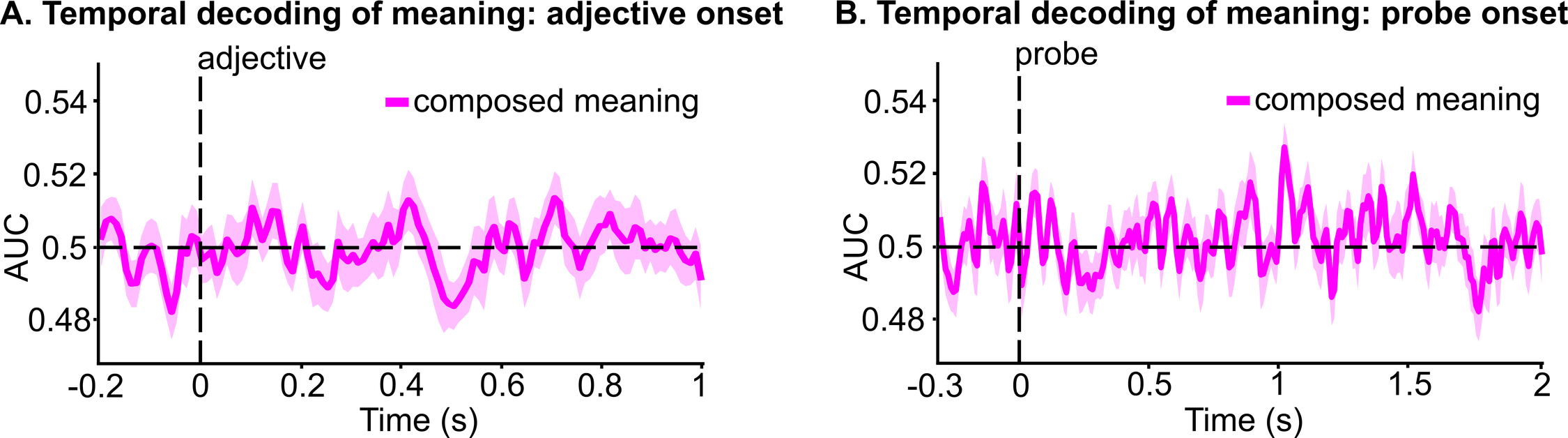

Supplement: S3 Fig — We trained estimators on phrases where the predicted composed meaning was “low” vs. “high” in 90% of the trials and computed the accuracy of the model in predicting the representation of the meaning “low” vs. “high” in the remaining 10% of the trials. For example, for the quality dimension, classes are: [0: bad] “### really bad,” “really ### bad,” “really really bad,” “### not good,” “not ### good,” “not not good,” “really not good,” “not really good”; and [1: good] “### really good,” “really ### good,” “really really good,” “### not bad,” “not ### bad,” “not not bad,” “really not bad,” “not really bad.” The composed meaning was derived from the behavioral results of Experiment 1. (A) Temporal decoding analyses time-locked to the onset of the adjective do not reveal any significant temporal cluster, suggesting that negation does not invert the representation of the adjective to that of its antonym (e.g., “bad” to “good”), as would be predicted by prediction (3) Inversion. (B) Temporal decoding analyses time-locked to the onset of the probe do not reveal any significant temporal cluster. For all panels: AUC = area under the receiver operating characteristic curve, chance = 0.5 (black horizontal dashed line); black vertical dashed lines indicate the presentation onset of the adjective in A and the probe in B; each line and shading represent participants’ mean ± SEM. (TIF) [file pbio.3002622.s003.tif]

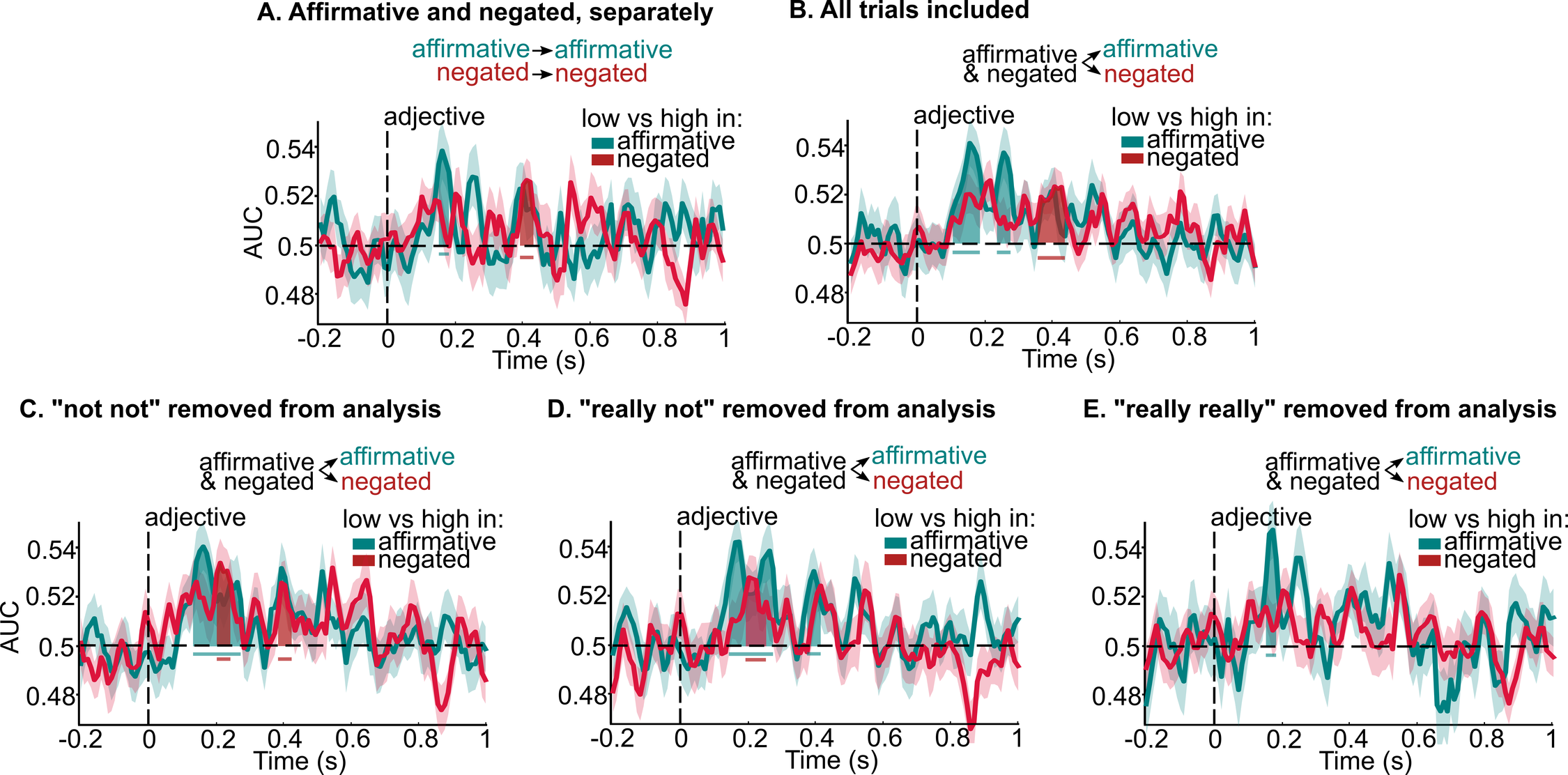

Supplement: S4 Fig — (A) We conducted a follow-up analysis where we trained and tested on “low” vs. “high” antonyms in affirmative and negated phrases separately, to further investigate lowering in decoding accuracy when representations are closer on the semantic scale, as predicted by the mitigation hypothesis for negated phrases. We found similar patterns to our main analysis. Results show that affirmative phrases (green line) are associated with significantly above-chance decoding accuracy between 150 and 190 ms (p = 0.026; green shading and horizontal solid line) from adjective onset. No significant above-chance decoding accuracy was found for negated phrases before approximately 400 ms from adjective onset (390 to 440 ms, p = 0.009; red shading and horizontal solid line). (B) We conducted a follow-up analysis where no trials were removed due to the feedback score. We found similar patterns to our main analysis. Results show that affirmative phrases (green line) are associated with significantly above-chance decoding accuracy between 100 and 190 ms and 230 and 280 ms from adjective onset (p = 0.001 and p = 0.032, respectively, green shading and horizontal solid lines). Negative phrases (red line) are associated with significantly above-chance decoding accuracy between 350 to 440 ms from adjective onset (p < 0.001, red shading and horizontal solid line). (C–E) We conducted a series of follow-up analyses where we removed one condition (i.e., 1 modifiers combination) at a time to evaluate its specific effect on adjective representation. (C) “not not” is removed from the analysis: Affirmative phrases (green line) are associated with significantly above-chance decoding accuracy between 130 and 280 ms from adjective onset (p < 0.001, green shading and horizontal solid line); negative phrases (red line) are associated with significantly above-chance decoding accuracy between 200 to 250 ms and between 380 to 430 ms from adjective onset (p = 0.011 and p = 0.049, red shading and horizontal so [file pbio.3002622.s004.tif]

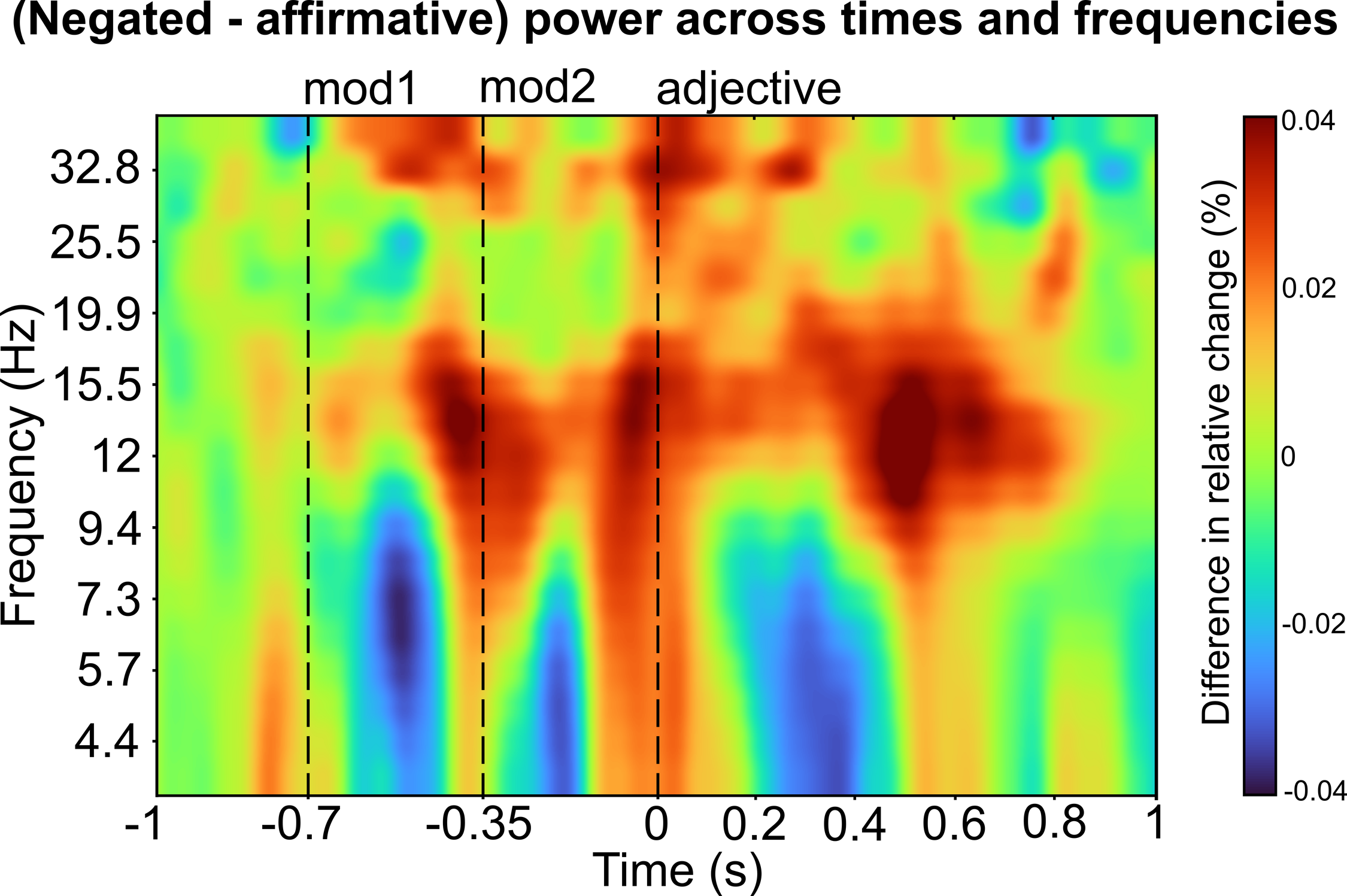

Supplement: S5 Fig — Time-frequency spectrum of the differences between negated and affirmative phrases averaged across all sensors and all participants. Frequencies are between 3.9 and 37.2 Hz, logarithmically spaced. Black vertical dashed lines indicate the presentation onset of each word: modifier1, modifier2, and adjective; colors indicate % differences in change relative to a baseline of −300 to −100 ms from the onset of word 1 (modifier1). (TIF) [file pbio.3002622.s005.tif]

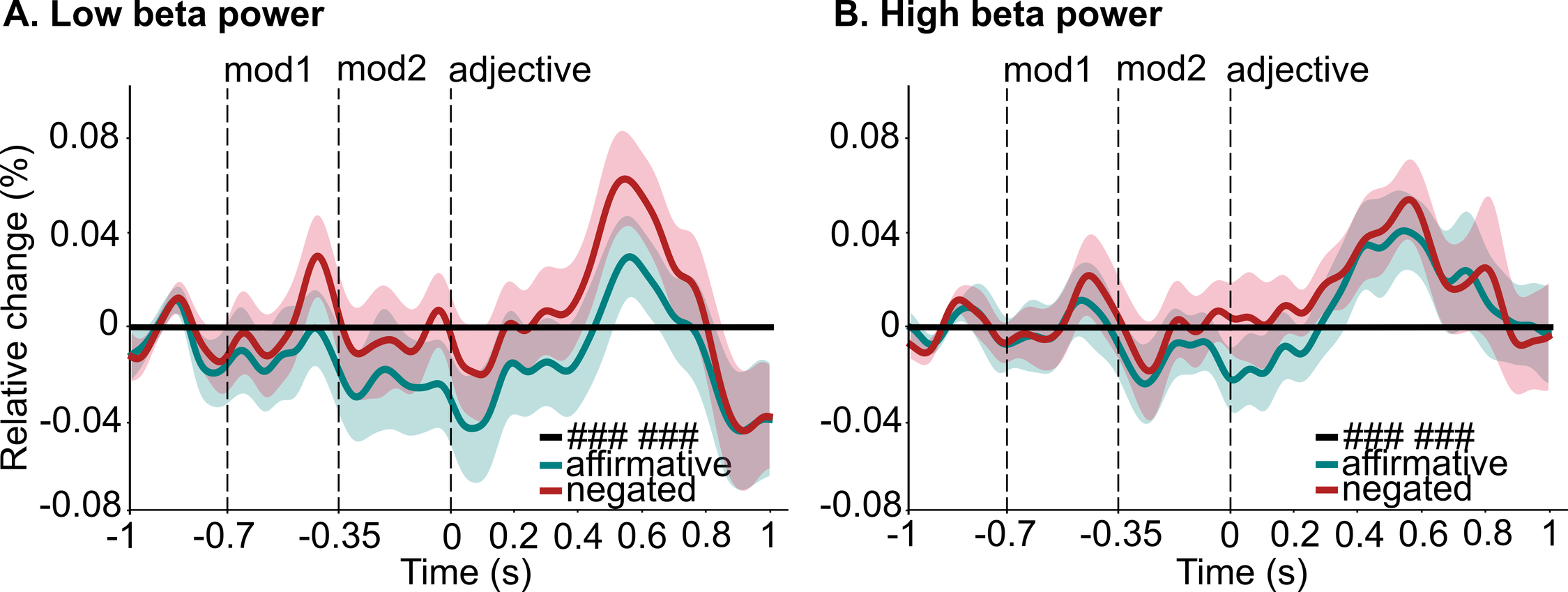

Supplement: S6 Fig — The mean beta power for the no modifier condition was subtracted from the mean beta power of affirmative and negated phrases, separately for low-beta (12–20 Hz, (A)) and high-beta (21–30 Hz, (B)). The horizontal solid black line represents the no modifier condition (i.e., ### ###) after subtraction (thus = 0), and the green and red lines represent beta power over time for affirmative and negated phrases, respectively. Relative change (%) was obtained by subtracting the mean of baseline values (−300 to −100 ms from the onset of word1) and dividing by the mean of baseline values. Black vertical dashed lines indicate the presentation onset of each word: modifier1, modifier2, and adjective; each line and shading represent participants’ mean ± SEM. (TIF) [file pbio.3002622.s006.tif]
