# Supplementary material for: Negation mitigates rather than inverts the neural representations of adjectives
Source: PLoS Biol. 2024 May 30;22(5):e3002622. doi: 10.1371/journal.pbio.3002622 (PMC11139306; doi:10.1371/journal.pbio.3002622)
Supplement: S1 Table — (DOCX) [file pbio.3002622.s007.docx]

**Table S1**

| **List of linguistic stimuli employed in Experiment 1 and replication (behavior)** | | | | | |
| --- | --- | --- | --- | --- | --- |
| ### ###  ### ###  ### ###  ### ###  ### ###  ### ###  ### ###  ### ###  ### ###  ### ###  ### ###  ### ###  ### really  ### really  ### really  ### really  ### really  ### really  ### really  ### really  ### really  ### really  ### really  ### really  really ###  really ###  really ###  really ###  really ###  really ###  really ###  really ###  really ###  really ###  really ###  really ### | small  big  cold  hot  ugly  beautiful  bad  good  sad  happy  slow  fast  small  big  cold  hot  ugly  beautiful  bad  good  sad  happy  slow  fast  small  big  cold  hot  ugly  beautiful  bad  good  sad  happy  slow  fast | really really  really really  really really  really really  really really  really really  really really  really really  really really  really really  really really  really really  ### not  ### not  ### not  ### not  ### not  ### not  ### not  ### not  ### not  ### not  ### not  ### not  not ###  not ###  not ###  not ###  not ###  not ###  not ###  not ###  not ###  not ###  not ###  not ### | small  big  cold  hot  ugly  beautiful  bad  good  sad  happy  slow  fast  small  big  cold  hot  ugly  beautiful  bad  good  sad  happy  slow  fast  small  big  cold  hot  ugly  beautiful  bad  good  sad  happy  slow  fast | not not  not not  not not  not not  not not  not not  not not  not not  not not  not not  not not  not not  really not  really not  really not  really not  really not  really not  really not  really not  really not  really not  really not  really not  not really  not really  not really  not really  not really  not really  not really  not really  not really  not really  not really  not really | small  big  cold  hot  ugly  beautiful  bad  good  sad  happy  slow  fast  small  big  cold  hot  ugly  beautiful  bad  good  sad  happy  slow  fast  small  big  cold  hot  ugly  beautiful  bad  good  sad  happy  slow  fast |
