# Supplementary material for: Negation mitigates rather than inverts the neural representations of adjectives
Source: PLoS Biol. 2024 May 30;22(5):e3002622. doi: 10.1371/journal.pbio.3002622 (PMC11139306; doi:10.1371/journal.pbio.3002622)
Supplement: S2 Table — Note that the condition with no modifiers (“### ###”) was only employed as a baseline condition in the time-frequency analysis. (DOCX) [file pbio.3002622.s008.docx]

**Table S2**

| **List of linguistic stimuli employed in Experiment 2 (MEG)** | | | | | |
| --- | --- | --- | --- | --- | --- |
| ### ###  ### ###  ### ###  ### ###  ### ###  ### ###  ### ###  ### ###  ### really  ### really  ### really  ### really  ### really  ### really  ### really  ### really  really ###  really ###  really ###  really ###  really ###  really ###  really ###  really ### | quiet  loud  cool  warm  dark  bright  bad  good  quiet  loud  cool  warm  dark  bright  bad  good  quiet  loud  cool  warm  dark  bright  bad  good | really really  really really  really really  really really  really really  really really  really really  really really  ### not  ### not  ### not  ### not  ### not  ### not  ### not  ### not  not ###  not ###  not ###  not ###  not ###  not ###  not ###  not ### | quiet  loud  cool  warm  dark  bright  bad  good  quiet  loud  cool  warm  dark  bright  bad  good  quiet  loud  cool  warm  dark  bright  bad  good | not not  not not  not not  not not  not not  not not  not not  not not  really not  really not  really not  really not  really not  really not  really not  really not  not really  not really  not really  not really  not really  not really  not really  not really | quiet  loud  cool  warm  dark  bright  bad  good  quiet  loud  cool  warm  dark  bright  bad  good  quiet  loud  cool  warm  dark  bright  bad  good |
