# Supplementary material for: Negation mitigates rather than inverts the neural representations of adjectives
Source: PLoS Biol. 2024 May 30;22(5):e3002622. doi: 10.1371/journal.pbio.3002622 (PMC11139306; doi:10.1371/journal.pbio.3002622)
Supplement: S3 Table — Each line represents a pairwise comparison between each pair of modifiers, for Experiment 1 (i.e., behavioral experiment, A) and its replication (B). p-Value and confidence intervals are adjusted for comparing a family of 9 estimates. Significant p-values are highlighted in bold. (DOCX) [file pbio.3002622.s009.docx]

**Table S3**

| **A. Experiment 1: Post Hoc Comparisons for RTs - Modifiers** | | | | | | | | | | | | | | | |
| --- | --- | --- | --- | --- | --- | --- | --- | --- | --- | --- | --- | --- | --- | --- | --- |
|  | | | | | | **95% CI for Mean Difference** | | | |  | | | | | |
|  | |  | | **Mean Difference** | | **Lower** | | **Upper** | | **SE** | **t** | | ***p*_tukey_** | |  |
| # # |  | # not |  | -0.120 |  | -0.214 |  | -0.026 |  | 0.030 |  | -3.956 |  | **0.003** |  |
|  |  | # really |  | -0.025 |  | -0.119 |  | 0.069 |  | 0.030 |  | -0.817 |  | 0.996 |  |
|  |  | not # |  | -0.110 |  | -0.204 |  | -0.016 |  | 0.030 |  | -3.642 |  | **0.009** |  |
|  |  | not not |  | -0.079 |  | -0.173 |  | 0.015 |  | 0.030 |  | -2.617 |  | 0.181 |  |
|  |  | not really |  | -0.087 |  | -0.181 |  | 0.007 |  | 0.030 |  | -2.876 |  | 0.096 |  |
|  |  | really # |  | -0.006 |  | -0.100 |  | 0.089 |  | 0.030 |  | -0.182 |  | 1.000 |  |
|  |  | really not |  | -0.090 |  | -0.185 |  | 0.004 |  | 0.030 |  | -2.988 |  | 0.071 |  |
|  |  | really really |  | 0.020 |  | -0.074 |  | 0.114 |  | 0.030 |  | 0.654 |  | 0.999 |  |
| # not |  | # really |  | 0.095 |  | 8.348e-4 |  | 0.189 |  | 0.030 |  | 3.139 |  | **0.046** |  |
|  |  | not # |  | 0.010 |  | -0.085 |  | 0.104 |  | 0.030 |  | 0.314 |  | 1.000 |  |
|  |  | not not |  | 0.041 |  | -0.054 |  | 0.135 |  | 0.030 |  | 1.338 |  | 0.920 |  |
|  |  | not really |  | 0.033 |  | -0.061 |  | 0.127 |  | 0.030 |  | 1.080 |  | 0.977 |  |
|  |  | really # |  | 0.114 |  | 0.020 |  | 0.208 |  | 0.030 |  | 3.774 |  | **0.005** |  |
|  |  | really not |  | 0.029 |  | -0.065 |  | 0.123 |  | 0.030 |  | 0.968 |  | 0.989 |  |
|  |  | really really |  | 0.140 |  | 0.045 |  | 0.234 |  | 0.030 |  | 4.610 |  | **< .001** |  |
| # really |  | not # |  | -0.086 |  | -0.180 |  | 0.009 |  | 0.030 |  | -2.825 |  | 0.110 |  |
|  |  | not not |  | -0.055 |  | -0.149 |  | 0.040 |  | 0.030 |  | -1.801 |  | 0.682 |  |
|  |  | not really |  | -0.062 |  | -0.157 |  | 0.032 |  | 0.030 |  | -2.059 |  | 0.502 |  |
|  |  | really # |  | 0.019 |  | -0.075 |  | 0.113 |  | 0.030 |  | 0.634 |  | 0.999 |  |
|  |  | really not |  | -0.066 |  | -0.160 |  | 0.028 |  | 0.030 |  | -2.171 |  | 0.426 |  |
|  |  | really really |  | 0.045 |  | -0.050 |  | 0.139 |  | 0.030 |  | 1.470 |  | 0.869 |  |
| not # |  | not not |  | 0.031 |  | -0.063 |  | 0.125 |  | 0.030 |  | 1.024 |  | 0.984 |  |
|  |  | not really |  | 0.023 |  | -0.071 |  | 0.117 |  | 0.030 |  | 0.766 |  | 0.998 |  |
|  |  | really # |  | 0.105 |  | 0.011 |  | 0.199 |  | 0.030 |  | 3.460 |  | **0.017** |  |
|  |  | really not |  | 0.020 |  | -0.074 |  | 0.114 |  | 0.030 |  | 0.654 |  | 0.999 |  |
|  |  | really really |  | 0.130 |  | 0.036 |  | 0.224 |  | 0.030 |  | 4.296 |  | **< .001** |  |
| not not |  | not really |  | -0.008 |  | -0.102 |  | 0.086 |  | 0.030 |  | -0.258 |  | 1.000 |  |
|  |  | really # |  | 0.074 |  | -0.020 |  | 0.168 |  | 0.030 |  | 2.435 |  | 0.266 |  |
|  |  | really not |  | -0.011 |  | -0.105 |  | 0.083 |  | 0.030 |  | -0.371 |  | 1.000 |  |
|  |  | really really |  | 0.099 |  | 0.005 |  | 0.193 |  | 0.030 |  | 3.271 |  | **0.031** |  |
| not really |  | really # |  | 0.082 |  | -0.013 |  | 0.176 |  | 0.030 |  | 2.693 |  | 0.152 |  |
|  |  | really not |  | -0.003 |  | -0.098 |  | 0.091 |  | 0.030 |  | -0.112 |  | 1.000 |  |
|  |  | really really |  | 0.107 |  | 0.013 |  | 0.201 |  | 0.030 |  | 3.529 |  | **0.013** |  |
| really # |  | really not |  | -0.085 |  | -0.179 |  | 0.009 |  | 0.030 |  | -2.806 |  | 0.115 |  |
|  |  | really really |  | 0.025 |  | -0.069 |  | 0.120 |  | 0.030 |  | 0.836 |  | 0.996 |  |
| really not |  | really really |  | 0.110 |  | 0.016 |  | 0.204 |  | 0.030 |  | 3.642 |  | **0.009** |  |
|  | | | | | | | | | | | | | | | |
|  | | | | | | | | | | | | | | | |

| **B. Replication of Experiment 1: Post Hoc Comparisons for RTs - Modifiers** | | | | | | | | | | | | | | |  |
| --- | --- | --- | --- | --- | --- | --- | --- | --- | --- | --- | --- | --- | --- | --- | --- |
|  | | | | | | **95% CI for Mean Difference** | | | |  | | | | |  |
|  | |  | | **Mean Difference** | | **Lower** | | **Upper** | | **SE** | **t** | | ***p*_tukey_** | |  |
| # # |  | # not |  | -0.122 |  | -0.265 |  | 0.020 |  | 0.046 |  | -2.669 |  | 0.162 |  |
|  |  | # really |  | -0.027 |  | -0.170 |  | 0.116 |  | 0.046 |  | -0.589 |  | 1.000 |  |
|  |  | not # |  | -0.115 |  | -0.258 |  | 0.028 |  | 0.046 |  | -2.512 |  | 0.229 |  |
|  |  | not not |  | -0.095 |  | -0.238 |  | 0.048 |  | 0.046 |  | -2.069 |  | 0.496 |  |
|  |  | not really |  | -0.123 |  | -0.266 |  | 0.019 |  | 0.046 |  | -2.692 |  | 0.153 |  |
|  |  | really # |  | -0.016 |  | -0.159 |  | 0.127 |  | 0.046 |  | -0.352 |  | 1.000 |  |
|  |  | really not |  | -0.123 |  | -0.266 |  | 0.020 |  | 0.046 |  | -2.689 |  | 0.154 |  |
|  |  | really really |  | 0.022 |  | -0.121 |  | 0.164 |  | 0.046 |  | 0.474 |  | 1.000 |  |
| # not |  | # really |  | 0.095 |  | -0.047 |  | 0.238 |  | 0.046 |  | 2.079 |  | 0.489 |  |
|  |  | not # |  | 0.007 |  | -0.136 |  | 0.150 |  | 0.046 |  | 0.157 |  | 1.000 |  |
|  |  | not not |  | 0.027 |  | -0.115 |  | 0.170 |  | 0.046 |  | 0.600 |  | 1.000 |  |
|  |  | not really |  | -0.001 |  | -0.144 |  | 0.142 |  | 0.046 |  | -0.023 |  | 1.000 |  |
|  |  | really # |  | 0.106 |  | -0.037 |  | 0.249 |  | 0.046 |  | 2.317 |  | 0.334 |  |
|  |  | really not |  | -9.364e-4 |  | -0.144 |  | 0.142 |  | 0.046 |  | -0.020 |  | 1.000 |  |
|  |  | really really |  | 0.144 |  | 0.001 |  | 0.287 |  | 0.046 |  | 3.143 |  | **0.046** |  |
| # really |  | not # |  | -0.088 |  | -0.231 |  | 0.055 |  | 0.046 |  | -1.922 |  | 0.599 |  |
|  |  | not not |  | -0.068 |  | -0.211 |  | 0.075 |  | 0.046 |  | -1.479 |  | 0.865 |  |
|  |  | not really |  | -0.096 |  | -0.239 |  | 0.046 |  | 0.046 |  | -2.102 |  | 0.473 |  |
|  |  | really # |  | 0.011 |  | -0.132 |  | 0.154 |  | 0.046 |  | 0.238 |  | 1.000 |  |
|  |  | really not |  | -0.096 |  | -0.239 |  | 0.047 |  | 0.046 |  | -2.100 |  | 0.475 |  |
|  |  | really really |  | 0.049 |  | -0.094 |  | 0.191 |  | 0.046 |  | 1.063 |  | 0.979 |  |
| not # |  | not not |  | 0.020 |  | -0.122 |  | 0.163 |  | 0.046 |  | 0.443 |  | 1.000 |  |
|  |  | not really |  | -0.008 |  | -0.151 |  | 0.135 |  | 0.046 |  | -0.180 |  | 1.000 |  |
|  |  | really # |  | 0.099 |  | -0.044 |  | 0.242 |  | 0.046 |  | 2.160 |  | 0.434 |  |
|  |  | really not |  | -0.008 |  | -0.151 |  | 0.135 |  | 0.046 |  | -0.178 |  | 1.000 |  |
|  |  | really really |  | 0.137 |  | -0.006 |  | 0.280 |  | 0.046 |  | 2.986 |  | 0.072 |  |
| not not |  | not really |  | -0.029 |  | -0.171 |  | 0.114 |  | 0.046 |  | -0.623 |  | 0.999 |  |
|  |  | really # |  | 0.079 |  | -0.064 |  | 0.221 |  | 0.046 |  | 1.717 |  | 0.736 |  |
|  |  | really not |  | -0.028 |  | -0.171 |  | 0.114 |  | 0.046 |  | -0.620 |  | 0.999 |  |
|  |  | really really |  | 0.117 |  | -0.026 |  | 0.259 |  | 0.046 |  | 2.543 |  | 0.214 |  |
| not really |  | really # |  | 0.107 |  | -0.036 |  | 0.250 |  | 0.046 |  | 2.340 |  | 0.320 |  |
|  |  | really not |  | 1.182e-4 |  | -0.143 |  | 0.143 |  | 0.046 |  | 0.003 |  | 1.000 |  |
|  |  | really really |  | 0.145 |  | 0.002 |  | 0.288 |  | 0.046 |  | 3.166 |  | **0.043** |  |
| really # |  | really not |  | -0.107 |  | -0.250 |  | 0.036 |  | 0.046 |  | -2.338 |  | 0.322 |  |
|  |  | really really |  | 0.038 |  | -0.105 |  | 0.181 |  | 0.046 |  | 0.826 |  | 0.996 |  |
| really not |  | really really |  | 0.145 |  | 0.002 |  | 0.288 |  | 0.046 |  | 3.163 |  | **0.043** |  |
|  | | | | | | | | | | | | | | |  |
|  | | | | | | | | | | | | | | |  |
