# Supplementary material for: Negation mitigates rather than inverts the neural representations of adjectives
Source: PLoS Biol. 2024 May 30;22(5):e3002622. doi: 10.1371/journal.pbio.3002622 (PMC11139306; doi:10.1371/journal.pbio.3002622)
Supplement: Attachment — Submitted filename: Response to Reviewers.pdf [file pbio.3002622.s010.pdf]

Response to Reviewers

We would like to thank the reviewers for their constructive and helpful comments and suggestions.

Following the reviewers' comments, we have substantially improved and expanded the Introduction, Results, and Discussion sections, and clarified the Methods section. In particular, we have clarified our approach and hypotheses in the Introduction are Results section, included the replication of Experiment 1 in the Results section, and performed seven new analyses that strengthen our results and which we have mentioned in the main text and included in the Supplementary Materials. Finally, we have further discussed similarities and differences between our behavioral and MEG results in the Discussion.

In the following, we present point-by-point responses, each followed by the changes/additions and the corresponding page numbers in the manuscript. In addition, we have highlighted all changes in orange in the manuscript.

REVIEWS:

Reviewer #1:

This manuscript investigates the time course of negated phrases in an abstract semantic meaning context. The authors used a behavioral paradigm and an MEG study to show that negation does not simply invert the meaning of the word, but rather mitigates the meaning of a words. This study used a smart paradigm and combined this with nice decoding approaches. I have some questions and remarks.

We thank the reviewer for the positive assessment.

Questions regarding the consistency of the MEG and the behavioral findings.
1) In the behavioral findings you show that participant first interpret the word as written and only later revert the meaning. Was it investigated whether the representations in MEG show the same pattern? If this pattern couldn't be found, how do the authors interpret this?

Thank you for raising this important question. Yes, we performed similar analyses on both behavior and MEG data, and identified similarities and differences. We now better clarify the relation between the MEG results and the behavioral findings in the Discussion:

"Taken together, our behavioral and neural data jointly point to a mitigation rather than an inversion effect of negation at early semantic processing stages, and exclude the hypothesis according to which negation does not change the representation of antonyms. Specifically, these results show that initial interpretations and early neural representations of negated adjectives are similar to that of affirmative adjectives, but weakened. The comparison between MEG and behavioral results also reveals interesting differences. Behavioral data reveal that, in negated

phrases, participants later modify their initial interpretation towards, but never exactly as, the opposite meaning. Our MEG data do not show an inversion of adjective representation as a function of negation, at early or later lexico-semantic processing stages. Differences between our behavioral and neural results could be ascribed to the fact that the behavioral task had to be adapted to the MEG environment. In the behavioral experiment (and its replication), participants were continuously and explicitly indicating their interpretation, while in the MEG experiment they were required to make a decision on their interpretation only after the probe was presented (1850 ms after the adjective presentation, Fig.2B and Fig.4A), which could have hindered later effects of negation." [lines 613-626]

2) The behavioral findings of final word interpretation don't really speak in favor for mitigation, but rather are in line with the inversion (not a full inversion, but still inverted). How do the authors connect the finding of the behavior with the MEG?

Thank you. This point relates to the comment above. We now clarify that the behavior and the MEG results show partially different patterns, namely, unlike behavior, the MEG patterns do not show a reversal. We still believe that these elements fit a mitigation hypothesis: First, negation does not invert the neural representation of the adjective but rather mitigates antonyms' representation at early lexical-semantic processing stages. Second, behavioral data indicate that, while participants prefer the opposite half of the scale, their final response does not overlap on the scale with that of the affirmative antonyms. In other words, this behavioral reversal is only partial.

We now discuss this issue in the paragraph quoted above.

Question on the decoding approach

3) Hypothesis figure in 5. Why for the change interpretation wouldn't one be able to decode in analysis ii) the affirmative conditions anymore? In my opinion the 'changed' representation would simply add noise to the training data set. Therefore, you could still decode the affirmative (maybe less strongly though).

This is a good point. We agree that, under the "change" hypothesis, negation could change the representation of the adjective to a representation on another semantic scale (however, not to the inverse) and that such a representation could potentially share some features with the affirmative (e.g., *low* and *fair*). In this case, the affirmative adjective could possibly still be decodable, however less accurately. We have changed the expected outcomes in Fig.6A as suggested by the reviewer.

4) In all the decoding approaches for low versus high there are only 4 words in the low and 4 words in the high category. It is possible that some of the word-specific, potentially visual features end up in the decoding rather than having it based on valence? This promotes the decoding of

the negated to be in-line with the affirmative option which might only related to irrelevant features. This will make it more difficult to find anything in line with the inversion hypothesis (which is more what the behavior speaks for). To get around this, I would train on three of the word pairs and test on the last word pair (and cross-validate). In this way one is sure that the effects are not due to any early sensory processing.

This is a good observation. We agree that training on three semantic scales and testing on the fourth might reveal new results. However, adjectives in each semantic scale were presented in a blocked fashion (to reduce task difficulty). Therefore we believe that our data structure is not well suited to perform the analysis suggested by the reviewer, and the results could be confounded by block identity. Furthermore, as rightly suggested by the reviewer, we wanted to avoid decoding (or failing to decode) only semantic and word specificity of the antonyms. Testing the model on one specific semantic scale, while training on three different semantic scales, might raise a similar problem, wherein the model is not able to decode the specific lexico-semantic features of one scale. Employing words with variability in terms of visual features and using multiple scales in training and testing sets decreases the chances that word-specific features could emerge. Finally, the number of semantic scales that we included is a tradeoff between stimulus variability, repetition of the same stimulus within one condition - which is essential to achieve a reliable decoding accuracy - and experiment duration for attention maintenance. We added this to the Methods section:

"The number of semantic scales (4) represents a tradeoff between stimulus variability, number of stimuli within each condition - which is essential to achieve a reliable decoding accuracy -, and experiment duration for attention maintenance." [lines 777-779]

5) In the mitigation hypothesis, one would also expect that when tested and trained on affirmative condition the decoding should work better than when tested and trained on negated condition. This occurs because it is more difficult to decode when the features are closer in some abstract multi-dimensional space (which is assume for the mitigation). I wondered why this analysis was not pursued as it seems the most simple one.

We thank the reviewer for suggesting this analysis, which we now added as Supplementary Fig.S4A. Accuracies, calculated as suggested by the reviewer, are shown below:

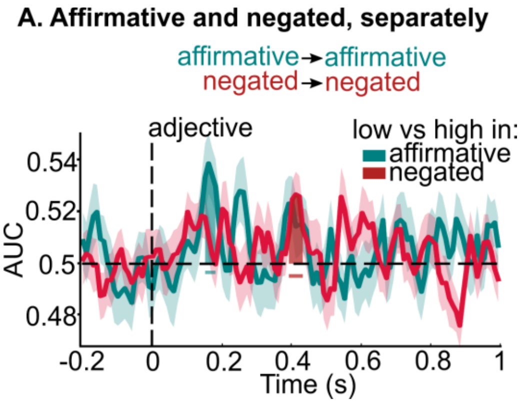

Here, AUC for affirmative is significantly above chance between 150 and 190 ms (*p* = 0.026, green shading) from adjective onset, and AUC for negated is significantly above chance from 390 to 440 ms from adjective onset (*p* = 0.009, red shading). This approach leads to patterns comparable to the ones we report in the manuscript, at similar time windows (see page 19). However, as the model is trained on approximately half of the trials (affirmative and negated separately), AUC can be noisier. Our decoding approach (ii) follows a similar reasoning as the one suggested by the reviewer, and it allows us to train the model on all trials: in the mitigation hypothesis, the representations of negated polar adjectives are weakened (or made less dissimilar along a binary semantic scale), thus leading to lower accuracy scores. We added the results of the analysis suggested by the reviewer in the Results section and the figure in the Supplementary Materials (Fig.S4A):

"A follow-up analysis where we trained and tested on low vs. high antonyms in affirmative and negated phrases separately shows similar results (Fig.S4A)." [lines 498-500]

"We conducted a follow-up analysis where we trained and tested on "low" vs. "high" antonyms in affirmative and negated phrases separately, to further investigate lowering in decoding accuracy when representations are closer on the semantic scale, as predicted by the mitigation hypothesis for negated phrases. We found similar patterns to our main analysis. Results show that affirmative phrases (green line) are associated with significantly above-chance decoding accuracy between 150 and 190 ms (p = 0.026; green shading and horizontal solid line) from adjective onset. No significant above-chance decoding accuracy was found for negated phrases before ~400 ms from adjective onset (390 to 440 ms, p = 0.009; red shading and horizontal solid line)." [Fig.S4, legend]

6) If you believe that there is a linear space on which the features can be mapped, why not approach the decoding as a regression rather than a binary feature. Then you could map everything in one go.

Thank you for this suggestion. We agree that a regression analysis (by fitting a ridge classifier, for example) would be an appropriate method to capture representational changes as a function of each modifier, and that such analysis would expand our conclusions and shed light on subtle representational differences. We have performed this analysis but we failed to find any effects. We believe that this is because this approach would require measuring exactly *where* participants locate the interpretation of *each* phrase on the binary semantic scale (e.g., "really really good" = 8, "really ### good" = 7 etc.), as the semantic scale might be neither linear nor represented identically across participants. Our design does not allow us to perform this type of analysis, as we did not collect participants' interpretations but only whether the interpretation they came up with corresponded to the probe we presented. Furthermore, we are not confident that 32 trials per condition would be sufficient to achieve robust results. A study with more trials per condition (and longer experiment time) which collects participants' specific interpretations could shed light onto more subtle representational differences that go beyond the sole effect of negation (e.g., "really not" vs. "not really").

Questions on design/method choices

7) Why are the words for the MEG and behavior different?

Similar to the behavioral experiment, in the MEG experiment we aimed to use semantic scales across different cognitive and sensory dimensions to avoid any bias and increase variability. For the MEG experiment, we reduced the number of trials due to time constraints (in our experience, MEG data quality is greatly reduced in quality for sessions longer than 90 minutes). We removed two dimensions in order to avoid removing trials per condition. We then balanced the remaining dimensions to represent three sensory systems (vision, audition, touch), and include one abstract dimension (quality). Words were also better controlled for lexical features (see Methods). We clarified this in the Methods section:

"Similar to Experiment 1, adjectives were selected for being antonyms (and contraries) in the following cognitive or sensory dimensions (touch, audition, vision): quality ("bad", "good"), temperature ("cool", "warm"), loudness ("quiet", "loud"), and brightness ("dark", "bright")." [lines 774-777]

8) Wouldn't it make sense to also analyze the data taking the 'not not' out? I understand you had it original because of the factorial design, but if for the participants it is so different one cannot really treat is as such (as also supplementary figure 2 shows).

We thank the reviewer for pointing to the specific effect of "not not". We agree that "not not" is not a frequent combination in natural language and its cognitive and linguistic representations are not well understood (e.g. Schiller et al. 2017, *Solving the problem of double negation is not impossible: electrophysiological evidence for the cohesive function of sentential negation. Language, Cognition and Neuroscience*). Both our behavioral and MEG analyses rely on our experimental design, where negated phrases are phrases where the modifier "not" is present, regardless of the participant's final interpretation, which we analyze in a second stage in the behavioral studies (Fig.2B and Fig.3B). Following the reviewer's suggestion, we have now performed the analysis for Fig.6C after removing phrases with "not not". To strengthen the conclusions, we also performed the same analysis after removing phrases with "really not" or "really really" which, similarly to "not not", are phrases with two modifiers, one negated and one affirmative. The comparison between these three data subsets allows us to speculate about the specific effect of "not not" while avoiding the confound emerging from differences in the number of trials per condition (i.e., a full dataset vs. a partial dataset). Decoding accuracies are shown below (Supplementary Fig.S4):

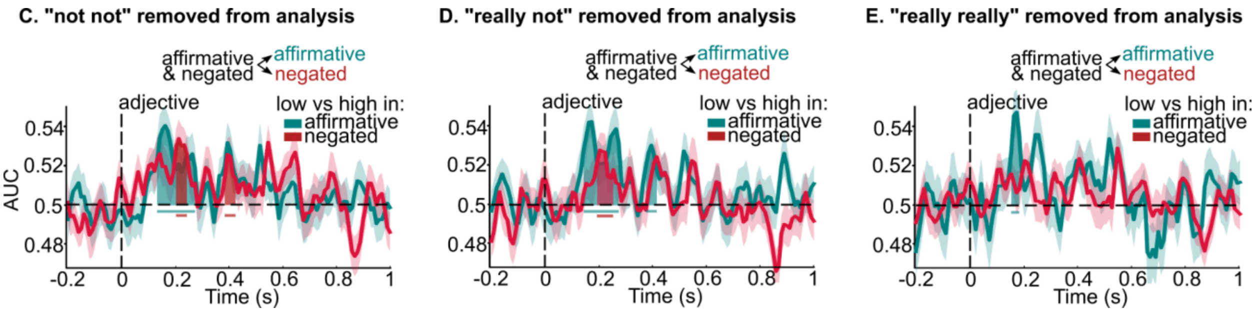

When “not not” is removed from the analysis (C), AUC for affirmative phrases is significantly above chance between 130 and 280 ms from adjective onset (p < 0.001, green shading), and AUC for negated phrases is significantly above chance between 200 to 250 ms and between 380 to 430 ms from adjective onset (p = 0.011 and p = 0.049, red shading). When “really not” is removed from the analysis (D), AUC for affirmative phrases is significantly above chance between 140 and 280 ms and between 370 and 420 ms from adjective onset (p = 0.001 and p = 0.038, green shading), and AUC for negated phrases is significantly above chance between 190 and 260 ms from adjective onset (p = 0.009, red shading). Finally, when “really really” is removed from the analysis (E), AUC for affirmative phrases is significantly above chance between 150 and 190 ms from adjective onset (p = 0.025, green shading), and AUC for negated phrases is never significantly above chance.

These results suggest that, when phrases with “not not” are removed, the mitigation effect is less strong, i.e., differences between low and high adjectives in negated phrases are significantly decodable at early representation stages (i.e., red shading in C). This result mirrors the results of the analysis where phrases with “really not” are removed (i.e., red shading in D), suggesting that “not not” and “really not” have similar mitigation effects. Conversely, when phrases with “really really” are removed, low and high adjectives in negated phrases are not significantly decodable, suggesting that “really really” does not have mitigation effects, as expected. Accordingly, we also observe that differences between low and high adjectives in affirmative phrases (i.e., green shading in E) are decodable for shorter time windows compared to C and D.

While these comparisons are based on the removal of one condition, as opposed to focusing on the direct effects of one specific condition (which would require more trials), these results suggest that “not not” also mitigates the representation of adjectives. While participants might explicitly know that two “not” cancel each other out (see Fig.3C), at early processing stages a double negation acts as a mitigator. We have added this observation to the Results section and the corresponding figure to the Supplementary Materials (Fig.S4):

“(“not not” was included to achieve a full experimental design, even if it is not a frequent combination in natural language. See Fig.S4C, Fig.S4D and Fig.S4E where we speculate that two “not”, i.e., double negation, do not cancel each other out but rather have mitigation effects similar to that of “really not”)” [lines 788-791]

“We conducted a series of follow-up analyses where we removed one condition (i.e., one modifiers combination) at a time to evaluate its specific effect on adjective representation. ***C****. “not not” is removed from the analysis: affirmative phrases (green line) are associated with significantly above-chance decoding accuracy between 130 and 280 ms from adjective onset ($p < 0.001$, green shading and horizontal solid line), negative phrases (red line) are associated with significantly above-chance decoding accuracy between 200 to 250 ms and between 380 to 430 ms from adjective onset ($p = 0.011$ and $p = 0.049$, red shading and horizontal solid lines).* ***D.*** *“really not” is removed from the analysis: affirmative phrases (green line) are associated with significantly above-chance decoding accuracy between 140 and 280 ms and between 370 and 420 ms from adjective onset ($p = 0.001$ and $p = 0.038$, green shading and horizontal solid lines), negative phrases (red line) are associated with significantly above-chance decoding accuracy between 190 to 260 ms from adjective onset ($p = 0.009$, red shading and horizontal solid lines).* ***E.*** *“really really” is removed from the analysis. affirmative phrases (green line) are associated with significantly above-chance decoding accuracy between 150 and 190 ms from adjective onset ($p = 0.025$, green shading and horizontal solid line), no significant above-chance decoding accuracy was found for negated phrases. Overall, these results suggest that “not not” and “really not” have similar mitigation effects. Conversely, and as expected, “really really” does not have mitigation effects on adjective representation.” [Fig.S4, legend]*

9) Why was decoding limited to only correct trials? This might force the analysis to only be focused on trials where the decoding was in line with the experimentally induced representation of the negated items.

Thank you for the reminder to address this. In the MEG experiment, feedback is provided after the probe - which is presented 1850 ms after the adjective onset - and exclusively relates to the comparison between the probe and the participant’s interpretation. We introduce this task to keep participants engaged and to avoid them responding without actually extracting the meaning of the phrase or paying attention. Following the reviewer’s suggestion, we changed the wording from “accuracy” to “feedback score” and we clarified that the feedback was provided only after the probe was presented:

“Feedback consisted of a green or red cross, to which 1 and 0 was assigned to compute the average feedback score.” [lines 319-320]

“In the behavioral experiment (and its replication), participants were continuously and explicitly indicating their interpretation, while in the MEG experiment they were required to make a decision on their interpretation only after the probe was presented (1850 ms after the adjective presentation, Fig.2B and Fig.4A), which could have hindered later effects of negation.” [lines 623-626]

Thus, we believe that we did not affect the initial representation of the adjectives by introducing feedback.

We thank the reviewer for suggesting this analysis, which we have now performed to verify that similar patterns are observable when all trials are included. Decoding accuracies are shown below (Supplementary Fig.S4):

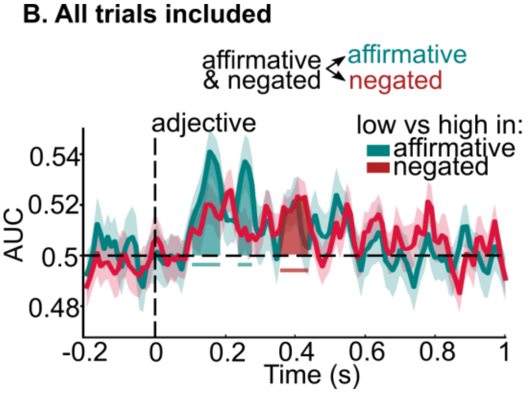

Results show that affirmative phrases (green line) are associated with significantly above-chance decoding accuracy between 100 and 190 ms and 230 and 280 ms from adjective onset (*p* = 0.001 and *p* = 0.032, green shadings). Negated phrases (red line) are associated with significantly above-chance decoding accuracy between 350 to 440 ms from adjective onset (*p* < 0.001, red shading). This approach leads to patterns comparable to the ones we report in the manuscript, at similar time windows. We have added these results in the Results section and the corresponding figure in the Supplementary Materials (Fig.S4):

"Furthermore, the analysis including all trials, regardless of feedback score, also shows similar results (Fig.S4B)." [lines 500-501]

"We conducted a follow-up analysis where no trials were removed due to the feedback score. We found similar patterns to our main analysis. Results show that affirmative phrases (green line) are associated with significantly above-chance decoding accuracy between 100 and 190 ms and 230 and 280 ms from adjective onset (p = 0.001 and p = 0.032 respectively, green shading and horizontal solid lines). Negative phrases (red line) are associated with significantly above-chance decoding accuracy between 350 to 440 ms from adjective onset (p < 0.001, red shading and horizontal solid line)." [Fig.S4, legend]

Questions on interpretation

10) In my opinion the 'replication' which is now the supplementary should be the main figure of the behavioral paper. If feedback was given what is the right answer in the original study you did not show how negation is represented, merely how participants can learn to follow instructions. It is good that the main patterns replicate, but I feel that now it is deceiving to put the effect so strongly as they appear in figure 1.

We understand the concern and appreciate the suggestion. As per the reviewer's argument, we have now moved the replication experiment to the main text as a main figure (now Fig.3). We agree that the replication experiment is less biased by feedback, however we are reassured by

the fact that the effect of negation does not markedly change with or without feedback, and it is actually more evident when feedback is removed (Fig.3B and Fig.3D). This similarity across experiments is now even clearer when the two figures are located in the main text, one after the other, as suggested by the reviewer.

11) I don't really see how you can define accuracy here. Depending on the context 'not bad' could feel closer to 'good' or closer to 'bad'. So, given that here there is no context how can you ever provide a definition of accuracy? This is a problem for the original behavioral study (especially as conclusion are drawn based on this). But it is the same issue with the MEG. How can you provide feedback on a 1-8 scale on how to interpret these abstract phrases? I also wonder whether this feedback forces people to represent the information in a specific manner that is forced by the experimenter. In the behavioral data that is now checked, but in the MEG not so this could bias the decoding.

This is an interesting and important point. We concur that accuracy might not be easy to define. We have changed the wording to “feedback score” throughout the text and figures, for all three experiments, and added more explanation where needed, e.g.:

“Feedback consisted of a green or red cross, to which 1 and 0 was assigned to compute the average feedback score.” [lines 319-320]

The feedback in both experiments was introduced to keep participants engaged with the task and to prevent them from responding without actually doing the task (i.e., extracting the meaning of the phrase) or attending to each trial. Ensuring that participants are engaged with the task is of the utmost importance, especially for MEG data collection. While this is only a proxy for accuracy, we find that the comparison between feedback score of affirmative and negated phrases is still informative of processing cost, which is mainly measured through RTs. We have added this in the Methods section:

“To keep the task engaging, participants were required to indicate whether the probe number represented the meaning of the phrase on the scale (yes/no answer). The order of the yes/no response keys was swapped halfway through the experiment. Responses had no time limit. If matching (+/- one step on the scale from a likely predefined value), a green fixation cross was presented; if not, a red fixation cross was presented, and feedback was provided.” [lines 808-812]

We also want to note that the two behavioral tasks show that adding or removing feedback does not change the conclusions of the experiment and that, thus, a similar outcome should be expected for the MEG data. In the MEG task, participants did not know what the probe was going to be until 1850 ms after the adjective onset. Our analyses focus on this time window, which should not be task-biased and should instead capture representation unfolding.

12) Is beta necessarily inhibition or could it also be something else? Beta has also been implied generally for feedback, not necessary for negative feedback.

Thank you for reminding us of this important issue. Beta-power modulation has been associated with multiple aspects of language processing, including semantic memory and syntactic binding, as well as feedback processing. We have further clarified this point in the Discussion:

"In addition to subserving motor processing, beta-power modulation (12-30 Hz) has been associated with attention and expectancy violation and with multiple aspects of language processing, such as semantic memory and syntactic binding, as well as feedback processing ([35,75–78]; for a review, see [57,79])." [lines 647-650]

In this study, we did not consider the possibility that differences between affirmative and negated phrases in the beta band could be due to feedback processing because we analyzed the time window right after the adjective onset and before the presentation of the probe, after which feedback was provided. We apologize if this was unclear, we have now made the timing of the task and the analyses more explicit in the Discussion:

"In the behavioral experiment (and its replication), participants were continuously and explicitly indicating their interpretation, while in the MEG experiment they were required to make a decision on their interpretation only after the probe was presented (1850 ms after the adjective presentation, Fig.2B and Fig.4A), which could have hindered later effects of negation." [lines 623-626]

13) In the conclusion it is stated that the behavioral effect shows that the negation never inverts the meaning of the word. While I agree it shows that it never completely inverts the meaning, one cannot state there is no inversion at all. It almost seems to suggest that the behavioral effects of the final decision support the MEG results, which I find quite a strong interpretation gives that in the MEG the negation does not cause a negated interpretation, but in the behavior it does. I think the trajectories have a good story that the initial representation matches the affirmative adjective, but for the final decision I think it simply doesn't match.

We agree that the behavioral results show that the interpretation of adjectives is closer to the affirmative antonym, as opposed to the affirmative adjective - although never overlapping (as demonstrated by final interpretations and beta values, Fig.2B and Fig.2D). We think that this remains an important result that argues clearly against the inversion hypothesis as formulated in Fig.5A. We have now clarified in the Discussion that, while the MEG data strengthen and extend the hypothesis that negation does not invert the lexical-semantic representation of adjectives within the first 600 ms but rather mitigates it, we do not find support that the representation of adjectives is inverted at later time windows, as found for the behavioral data:

"Behavioral data reveal that, in negated phrases, participants later modify their initial interpretation towards, but never exactly as, the opposite meaning. Our MEG data do not show an inversion of adjective representation as a function of negation, at early or later lexico-semantic processing stages. Differences between our behavioral and neural results could be ascribed to the fact that the behavioral task had to be adapted to the MEG environment. In the behavioral experiment (and

its replication), participants were continuously and explicitly indicating their interpretation, while in the MEG experiment they were required to make a decision on their interpretation only after the probe was presented (1850 ms after the adjective presentation, Fig.2B and Fig.4A), which could have hindered later effects of negation." [lines 618-626]

We have also further stressed the absence of evidence supporting the inversion hypothesis at later time windows for MEG data in the Results section:

"No significant difference between decoding accuracy of affirmative and negative phrases was found for later time windows (500-1000 ms from adjective onset, $p > 0.05$)." [lines 496-498].

Other

14) In the abstract a conclusion is made on the MEG, but it would be nice to spend just a few words on how this conclusion could be reached.

We thank the reviewer for this good suggestion. We have now expanded the abstract to better guide the reader:

"Decoding analyses of neural activity further reveal significant above chance decoding accuracy for negated adjectives within 600 ms from adjective onset, suggesting that negation does not invert the representation of adjectives (i.e., "not bad" represented as "good"); furthermore, decoding accuracy for negated adjectives is found to be significantly lower than that for affirmative adjectives. Overall, these results suggest that negation mitigates rather than inverts the neural representations of adjectives."

15) Line 671. What does the swapping of yes/no means. Is it just the response buttons or something else? This wasn't clear.

Thank you, we have clarified that we swapped the order of the yes/no response keys:

"The order of the yes/no response keys was swapped halfway through the experiment." [lines 809-810].

Reviewer #2:

The article tests how combinatoric representation of negation and adjectives operates in both behavioral and MEG experiments. The results from RT and mouse trajectory show that negated adjectives do not change into the opposite meaning (e.g. "not good" doesn't become "bad".). The MEG decoding analysis shows that negation mitigates the meaning of antonyms, ruling out other

three possible hypotheses (i.e., no change, inversion, change). The authors also report that beta power was increased in negation, in line with the suppression effect in the previous research. Overall, I found the research was conducted in a rigorous way. It is also valuable to see what kind of combinatoric processes relating to negation occur in our brain. However, the overall analysis is unclear to address the research question. Please find below for my comments.

We thank the reviewer for appreciating the rigor of the work.

Major comments:

I was confused when I read the plots since it's not really straightforward and clear what conditions the authors compared. The authors had an elegant design of the experiment. However, it is not clear to me how they compare the conditions they have. For example, in Fig.4C, they showed decoding accuracy of negation with 1 and 2 modifiers. "1 modifier" could mean "### really good", "### not good", "really ### good", "not ### good". Maybe also including an example explicitly in the plot might make it clearer.

We thank the reviewer for catching this, and we apologize for any misunderstandings. We have now clarified this in the figure legends, by adding all possible combinations:

"1 modifier: "really ###", "### really", "not ###", "### not"; 2 modifiers: "really really", "really not", "not really", "not not"." [Figure 5 and Figure S2, legend]

Also, I am concerned about the effect of placeholder "###". For my intuition, it would be weird to read the stimuli like "really ### good". I think the authors should have some additional analysis to rule out any potential effect from the placeholder. For example, should there be no difference when comparing "### really" vs. "really ###" if the placeholder does not affect the combinatoric processes?

Thank you for raising this important point. Previous foundational psycholinguistics studies looking at linguistic representation and composition have employed visual strings as placeholders to mark the absence of linguistic information (e.g., Bemis and Pylkkänen 2011; Westerlund et al., 2015). We relied on this earlier methodology when building our stimuli.

However, we agree with the reviewer that an in-depth analysis of each modifiers' combination extends the results of this study. Consequently, we have added a comprehensive table with all pairwise comparisons to the Supplementary Materials and mentioned it in the Results section. We confirm that no significant difference in RTs was found, e.g., between "### really" vs. "really ###" and "### not" vs. "not ###":

"see Table S3A for pairwise comparisons between each pair of modifiers" [lines 186-187]

“see Table S3B for pairwise comparisons between each pair of modifier” [lines 260-261]

The overall article may mainly focus on the comparison "really not good" vs. "really really good", but why not just compare "really good" vs. "not good"? I was not really sure

We thank the reviewer for raising this point, and we agree that such design would have been simpler. However, our behavioral study allows for a more fine-grained characterization of how negation operates on meaning along the semantic scale (Fig.2B and Fig.3B). We wanted to draw parallel conclusions between behavioral and MEG results, and we thus employed the same experimental design. This design has also allowed us to observe differences in how negation is processed as a function of complexity (Fig.4C). Finally, in the same way we have multiple adjective dimensions to increase stimulus variability and draw more general conclusions beyond the single word, we also wanted to diversify the modifiers to be able to measure how negation affects adjective representation above and beyond the specific effects of the words “really” and “not”. We have clarified this point in the Methods section:

“Each of the 12 adjectives was preceded by each of the nine possible combinations of modifiers: “### ###”, “### really”, “really ###”, “### not”, “not ###”, “really not”, “not really”, “really really” and “not not”, to diversify modifiers’ sequences and measure how negation affects adjective representation above and beyond the specific effects of the words “really” and “not”” [lines 725-729]

if the experiments really needed stimuli with placeholder if they didn't show full analysis on those stimuli. Since the authors have included stimuli with "###", I think the authors should also analyze them or specify them explicitly in the text/plots.

Thank you for the suggestion. We introduced stimuli with placeholders to manipulate complexity (e.g., “### not” vs. “really not”). The results of the decoding analysis for complexity are shown in Fig.5C and Fig.S2. We have clarified that complexity was manipulated through the placeholder in the figure legend:

“1 modifier: “really ###”, “### really”, “not ###”, “### not”; 2 modifiers: “really really”, “really not”, “not really”, “not not”.” [Figure 5 and Figure S2, legend]

Overall, it is sometimes not straightforward how some analyses address the research questions. For example, how does the analysis in Fig. 4A and 4B address the neural processes of negation? It seems like the comprehensive design is used somewhat incompletely in terms of the contrasts used in certain analyses (when pooling over different modifier conditions. In general, the steps between questions, hypotheses, predictions (of the results) and analyses could be more clearer.

We thank the reviewer for requesting important clarification for the reader. We have now improved this in the revised Results section and referred to supplementary Table S1 and S2 more pointedly, where the specific contrasts used are made explicit:

"The MEG analyses, using largely temporal and spatial decoding approaches [48], comprise four incremental steps: (1) we first identify the temporal correlates of simple word representation (i.e., the words "really" and "not" in the modifier position, and each pair of scalar adjectives in the second word position, i.e., the head position; see Table S2); (2) we test lexical-semantic representations of adjectives over time beyond the single word level, by entering low ("bad", "cool", "quiet" and "dark") and high ("good", "warm", "loud" and "bright") antonyms in the same model (adjectives in purple vs. orange in Table S2). We then test the representation of the negation operator over time (modifiers in green vs. red in Table S2); (3) we then ask how negation operates on the representation of adjectives, by teasing apart four possible mechanisms (i.e., No effect, Mitigation, Inversion, Change; adjectives in purple vs. orange for modifiers in green and red separately in Table S2); (4) we explore changes in beta power as a function of negation (motivated by the literature implicating beta-band neural activity in linguistic processing)." [lines 324-335]

"After establishing that single words' features can be successfully decoded in sensible time windows (see Fig.4), we moved beyond single word representation and clarified the temporal patterns of adjective and negation representation independently from their interaction and identified temporal windows where to expect changes in adjective representation as a function of negation." [lines 367-370]

Minor comments:

Introduction:
Ln 72-75: It's not clear to me here what you mean: "the building blocks" and "stem from more subtle inferential meaning". The examples given here could be explained further.

We thank the reviewer for highlighting this. We have changed the examples and clarified what we mean with "building blocks" and "subtle inferential meaning":

"The composed meanings can be extracted in relatively straightforward ways, such as by sequentially combining individual meanings of words and phrases (e.g., "this theory is correct") or stem from more subtle inferential processes, where further operations are required to achieve understanding (e.g., "this theory is not even wrong", meaning "this theory is incoherent")." [lines 72-76]

Ln 119: why is the decoding approach applied?

We have now clarified this in the Introduction:

“Mouse tracking and decoding approaches allow us to quantify and compare dynamic changes in participants’ interpretations and neural representations of adjectives over time (e.g., [45,46]).” [lines 123-125]

Results/Methods

Exp1

Reaction times

Ln 170-171: Participants were faster for affirmative phrases (e.g. really really good) than for negated phrases (e.g. really not good). What about "not really good" and also "really good" vs. "not good"? I also wonder if there is a difference in "### really" vs. "really ###" and "### not" vs. "not ###". I would expect no difference here. It would be great if the authors could provide pairwise comparisons here.

Thank you. We have added a comprehensive table with all pairwise comparisons to the Supplementary Materials and mentioned it in the Results section. We confirm that no significant difference in RTs was found between "### really" vs. "really ###" and "### not" vs. "not ###":

“see Table S3A for pairwise comparisons between each pair of modifiers” [lines 186-187]

“see Table S3B for pairwise comparisons between each pair of modifier” [lines 260-261]

Exp 2

Ln 736: Please include the interval of the number of removed components.

We have now included the mean and standard deviation of the number of removed components.

“FastICA was used to decompose the signal into 20 independent components, to visually inspect and remove artifacts related to eye-blinks, heartbeat, and external noise sources (removed components across blocks and participants: mean = 5.98, SD = 1.73).” [lines 877-880]

Ln 752: Why was the noise covariance matrix estimated from the 300ms before the onset of the first word? And up to when?

The noise covariance matrix was estimated from 300 ms up to 0 ms from the onset of the first word. This time corresponds to the pre-stimulus baseline and was chosen to be as far as possible from the feedback presentation, while leaving enough time samples for baseline correction. We clarified its length in the Methods section:

“A noise covariance matrix was estimated from the 300 ms before the onset of the first word up to the onset of the first word presentation.” [lines 894-896]

Ln 774-775: So does it mean that there is a threshold for the number of trials to decide when you use 5-fold/10-fold cross-validation? Is it possible to report a number here?

We used a 10 fold cross-validation when we employed all of the stimuli (e.g., decoding of antonyms and negation in Fig.5A and 5C and decoding approach (ii) in Fig.6C). We used a 5-fold cross-validation when we employed a subset of all the stimuli (e.g., decoding of modifier *really* vs *not* and decoding of adjective as letter string in Fig.4B and 4C, which included half of the data and one quarter of the data, respectively). We specified which analyses employed a 5-fold or a 10-fold cross-validation approach in the Methods section:

“We then employed a 5-fold (for analyses in Fig.4B and Fig.4C) or 10-fold stratified cross-validation (for analyses in Fig.5A, Fig.5C, and Fig.6C) that fitted the estimator to 80% or 90% of the epochs and generated predictions on 20% or 10% of the epochs, while keeping the distributions of the training and test set maximally homogeneous.” [lines 917-920]

Ln 796/Ln 809 / Ln 351: maybe state explicitly what train/test set were used for both decoding approaches. The authors only mentioned train/test for the first approach, but not the second, though I assume the train set would include "really really bad/good" and "really not bad/good".

Thank you. We have added a reference to Supplementary Table S2 to make the train/test sets more explicit:

“Approach (i) train set: affirmative phrases (in green in Table S2); test set: negated phrases (in red in Table S2).” [lines 938-939]

“Approach (ii) train set: affirmative and negated phrases together (in green/red in Table S2); test set: affirmative and negated phrases separately (in green and red in Table S2).” [lines 951-952]

Ln 817-819/Fig. 5A: Though it looks like the authors provided some kind of explanation here, I am still not clear why the prediction of (3) inversion would have the same prediction of (4)? Why not expect a reversed direction for negation?

Thanks for raising this, it is an important point. Prediction 3 and 4 differ in the outcome of decoding approach (i), with prediction 3 showing a reversed effect for negated phrases when the model is trained on affirmative (e.g., class 1: *good* vs. class 2: *bad*) and tested on negated (e.g., class 1: *not good=bad* vs. class 2: *not bad=good*). When including both affirmative and negated phrases in the training set (i.e., class 1: *good* and *not good=bad*), the model cannot learn the data features and thus cannot make reliable predictions (i.e., accuracy at chance).

Please note that, in response to Reviewer 1, we have updated the expected outcome of *change* for decoding approach (ii): under the “change” hypothesis, negation could change the representation of the adjective to a representation on another semantic scale (however not to the inverse of the affirmative) and that such representation could potentially share some features with the affirmative (e.g., *low* and *fair*). In this case, the affirmative adjective could possibly still be decodable, however less accurately. We have changed the expected outcomes in Fig.6A accordingly.

Fig.3: overall, I am not sure if these really reflect single word processing for modifiers. The authors might need to include control analysis of isolated adjective ("### ### good") and also basically pairwise comparisons for each condition. It was not clear why the condition "### ###" is not sufficient to serve as a baseline in all analyses (Ln 652-654).

The reviewer brings up an interesting point. Results in Fig.3B (now 4B) reflect single word processing for modifiers, as the two classes only differ in the modifier (e.g., “really ###” vs “not ###”) and all adjectives are included in each class, regardless of whether they are low or high. This figure has the purpose of highlighting two representative examples of pairwise word decoding, when words are modifiers. The figure serves as a “baseline” to show that single words can be decoded with relatively high accuracy (~70%). We have clarified this in the Results section:

“These results further show that single words can be decoded with relatively high accuracy (~70%)” [Lines 349-350]

Results in Fig.4A (now 5A) serve as “baseline” to identify the time window where lexical-semantic differences between antonyms can be decoded. We believe this is a more reliable result than what we would obtain using the condition "### ###" in isolation, given the difference in sample size (512 trials vs 64 trials) and taking into account the modifier variability which - we believe - makes our results more robust.

minor: maybe add lines/shape to highlight the significant timing. I found it's hard to target the significant timing in the plots.

Thank you. We have added horizontal solid lines to better highlight the significant timings for affirmative and negated phrases in Fig.6B, 6C, and Supplementary Figure S4.

Please also explain Fig. 3A in the text.

We have explained Fig.3A (now 4A) in text:

“The butterfly (bottom) and topography plots (top) in Fig.4A illustrate the grand average of the event-related fields elicited by the presentation of all words, as well as the probe, regardless of

condition. Results of decoding analyses performed on these preprocessed MEG data (after performing linear dimensionality reduction; see Methods) show that the temporal decoding of "really" vs. "not" is significant between 120 and 430 ms and between 520 and 740 ms from the onset of the first modifier (dark gray shading, $p < 0.001$ and $p = 0.001$) and between 90 and 640 ms from the onset of the second modifier (light gray shading, $p < 0.001$, Fig.4B)." [lines 338-344].

Fig. 3B: This figure is showing increased decoding accuracy from 50ms after the onset of the modifier. Which sensors contributed mostly to decoding? Topographies in Fig. 3A shows occipital effect consistent with the presentation of the visual stimuli. Could ### serve as a baseline for the visual effect vs combinatorics? How does the figure address questions on negation?

In temporal decoding analyses, before fitting the estimators, linear dimensionality reduction (principal component analysis, PCA) was performed on the channel amplitudes to project them to a lower dimensional space (i.e., to new virtual channels that explained more than 99% of the feature variance; see Data analysis). We have clarified this in the Results section:

"The butterfly (bottom) and topography plots (top) in Fig.4A illustrate the grand average of the event-related fields elicited by the presentation of all words, as well as the probe, regardless of condition. Results of decoding analyses performed on these preprocessed MEG data (after performing linear dimensionality reduction; see Methods) show that the temporal decoding of "really" vs. "not" is significant between 120 and 430 ms and between 520 and 740 ms from the onset of the first modifier (dark gray shading, $p < 0.001$ and $p = 0.001$) and between 90 and 640 ms from the onset of the second modifier (light gray shading, $p < 0.001$, Fig.4B)." [lines 338-344].

While Fig.4B serves to show that single words' features can be successfully decoded in expected time windows and with expected patterns, Fig.5C illustrates the effect of negation as a function of complexity (or combinatorics effects). We showed that, for phrases with two modifiers, negation is decodable for a longer time window compared to phrases with one modifier, both after adjective onset and after probe onset (Fig.S2).

Ln 261: significant time: 520-740ms How can you make sure this timing correlates with the first word but not with the stimulus in the second modifier position? The interstimulus intervals are quite short by design, so is it safe to attribute effects beyond the ISI to the first word? As visual stimuli are presented with such a short ISI, it's not surprising the evoked responses are mainly visual (see topographies in 3A). How does it affect the decoding? Once again, could the neutral modifier serve as a useful baseline for visual, not semantic/combinatoric activity?

We thank the reviewer for raising this point. Decoding analyses were run separately for "### really" vs. "### not" and "really ###" vs. "not ###". Thus, in these analyses there are never two modifiers, therefore the figure exclusively shows differences in neural representation between "really" and "not" when they are in the first modifier position (dark gray line) or second modifier position (light gray line). We have made this more clear in the figure legend:

(B) We estimated the ability of the decoder to discriminate "really" vs. "not" separately in the first and second modifier's position, from all MEG sensors. [Figure 4, legend].

Topographies in Fig.3A (now 4A) represent a grand-average of all trials, regardless of condition, which all include a visual component. This figure has the purpose of illustrating the raw signal used to perform targeted and condition-based multivariate decoding analyses. We have clarified this in the Results:

"The butterfly (bottom) and topography plots (top) in Fig.4A illustrate the grand average of the event-related fields elicited by the presentation of all words, as well as the probe, regardless of condition. Results of decoding analyses performed on these preprocessed MEG data (after performing linear dimensionality reduction; see Methods) show that the temporal decoding of "really" vs. "not" is significant between 120 and 430 ms and between 520 and 740 ms from the onset of the first modifier (dark gray shading, $p < 0.001$ and $p = 0.001$) and between 90 and 640 ms from the onset of the second modifier (light gray shading, $p < 0.001$, Fig.4B)." [lines 338-344].

In the previous behavioral task, the authors mentioned that the placeholder may induce some interference. I wonder if the authors could rule out the influence of "###" from the analysis.

By counterbalancing our experimental conditions (where ### is present both for affirmative and negated phrases), we aimed to rule out the selective effect of the placeholder. Fig.4B shows a very similar pattern for "really" vs "not" when the placeholder is presented or not before the modifiers, suggesting that the effect of the placeholder is minimal (if any). We agree with the reviewer that our interpretation of that specific behavioral result could be misleading; we have thus refrained from any speculation about the effect of ###, which might deserve further investigation in a dedicated experiment with an increased number of trials per condition, and a more specific design:

"suggesting that the placeholder "###" may induce some processing slow-down. To confirm this hypothesis, further research should investigate the specific effect of placeholders (e.g., "###" or "xkq") on word and phrase representation and semantic composition." [lines 187-189].

Also, the timing starting from 700ms is the timing for an adjective. How can the authors make sure that the effect is not from the adjective?

Fig.5A shows the decoding accuracy (purple line) of lexical-semantic differences between polar adjectives across all scales, where adjectives are presented at 700ms from the first modifier's onset. By pooling over all the modifiers, which are the same for both polar adjectives, we restrict our analysis to the representation of low vs high adjectives. We clarified this in the Results section:

“First, we selectively evaluated lexical-semantic differences between low (“bad”, “cool”, “quiet” and “dark”) and high (“good”, “warm”, “loud” and “bright”) adjectives, regardless of the specific scale (i.e., pooling over quality, temperature, loudness, and brightness) and by pooling over all modifiers.” [lines 370-373].

Fig. 3C: The figure shows the comparisons between antonyms. What conditions exactly do the authors compare here? For example, "### ### good" vs. "### ### bad"? "### really good" vs. "### really bad"? or collapsed the adjectives and analyzed them together?

Thank you for catching that, we included all the phrases that included each of the antonyms. We have specified this in the Results section:

“Pairs of antonyms from different scales (regardless of specific modifier) were similarly decodable between 90 and 410 ms from adjective onset” [lines 344-346]

Fig. 4C: I am confused about what conditions the authors exactly compare here.
1 modifier: ### really good vs. ### not good? really ### good vs. not ### good?
2 modifiers: "really really good" vs. "not not good"? or "really not good" vs. "not really good"?

We have clarified this in the figure legend of Fig.4C (now 5C)”

“(C) Decoding accuracy of negation over time, as a function of the number of modifiers (1 modifier: dark red line and shading; 2 modifiers: light red line and shading). 1 modifier: “really ###”, “### really”, “not ###”, “### not”; 2 modifiers: “really really”, “really not”, “not really”, “not not”.” [Figure 5, legend]

Ln327-330: The authors mentioned: these results suggest that the brain encodes negation every time a "not" is presented and maintains this information up to 720 ms after adjective onset. However, From Ln 327, they also found the analysis of probe shows that negation is once again significantly decodable between 230 and 930 ms from probe onset (i.e. almost 4 seconds after the first modifier). It looks like the effect is not just up to 720 ms, but even in the later stage up to the response. I wonder up to when after the onset of the modifier for decision?

We showed that the representation of negation is sustained up to 720 ms after adjective onset. We show a reactivation after the probe onset likely due to the task, where participants were required to indicate whether the probe number represented the meaning of the phrase on the scale. Thus we do not interpret this as a sustained representation of negation, or as negation being represented at two different stages, but rather as the representation of negation being *reinstated* for task purposes (i.e., after the presentation of the probe). We have clarified this in text:

“The same analysis time-locked to the onset of the probe shows that negation is once again significantly decodable between 230 and 930 ms after the probe, likely being reinstated when participants perform the task (Fig.S2).” [lines 415-417]

Ln 345: "(3) and (4)" should be "(2) and (3)"?

Thank you, we have amended this.

Fig. 6A: negated - affirmative: really not good - really really good? or not really good - really really good? or others?

We have now specified in the figure legend:

“Differences in low (12-20 Hz, red) and high (21-30 Hz, yellow) beta power over time between negated (i.e., “### not”, “not ###”, “really not”, “not really”, “not not”) and affirmative phrases (i.e., “### really”, “really ###”, “really really”).” [Figure 7, legend]

Fig. 6B: The authors mention that only 10 subjects had individual MRI-based source models (Ln 746). I wonder what's the reason for it and how serious of an issue that is in the current experiment.

Good question. We were able to collect MRIs of participants who agreed to come a second time for the MRI scan. MEG and MRI did not take place in the same session, thus only a subgroup of participants agreed to come a second time. We would like to mention that data acquisition for this study took place before the COVID-19 pandemic, and a subgroup of participants had moved elsewhere when in-person studies were again allowed, thus we could not collect their MRI. While we understand the concern of the reviewer, we followed a standardized practice for manually scaling and co-registering the “fsaverage” brain to the participant’s head-digitized shape and fiducials, so we do not think this is an issue.

Discussion

Ln 479-482: I am not sure why the processing of adjectives up to 700 ms in Fig.4C suggests negation is processed in parallel to the processing of the adjective, but not serially. Maybe specify explicitly what serial processing would be like here.

We thank the reviewer for the opportunity to better expose this point. We have expanded the text as follows:

“Overall, these data demonstrate that both scalar adjectives and negation are represented between 140 and 560 ms from adjective onset (compare Fig.5A and Fig.5C), suggesting that they are represented in parallel and not serially (i.e., one after the other; see [69,70] for related patterns in the context of negation + auxiliary verb and adjective + noun).” [lines 575-580]

The authors might discuss the time-frequency effects in more detail. In particular, they might want to discuss whether they could be specific to negation or if they might reflect other processes (e.g. expectations at the morpheme and phrasal level).

Thank you for pointing to this significant issue. We agree that beta-power modulation has been associated with other linguistic and non-linguistic processes, including attention and expectancy violation, semantic memory, and syntactic binding, as well as feedback processing. In this study we focused on differences between affirmative and negated phrases in the beta band. We have further clarified the involvement of beta in multiple language and other processes in the Discussion:

"In addition to subserving motor processing, beta-power modulation (12-30 Hz) has been associated with attention and expectancy violation and with multiple aspects of language processing, such as semantic memory and syntactic binding, as well as feedback processing ([35,75–78]; for a review, see [57,79])." [lines 647-650]

Reviewer #3:

The manuscript reports a study on the processing of negation which combines behavioral and neurophysiological data. Previous studies have addressed this issue mostly from a psycholinguistic perspective, showing that negation incurs processing costs and pointing to a two-stage processing mechanism, whereby the initial interpretation attributed to a negated adjective is closer to that of the adjective than to that of the antonym. This study therefore carries the potential to add interesting, novel and timely insights into negation processing. But most importantly, this study is not just about negation processing, since negation is used as a proxy to investigate a more basic, yet fundamental issue such as online meaning construction. And this is what makes this work interesting to a wide readership.

We thank the reviewer for their positive take on our work and for pointing to the broader significance of our results.

The results show that negated phrases elicit longer response times, both in the mouse-tracking and in the behavioral task performed during the MEG experiment. Moreover, both the initial and final interpretation of negated phrases such as "not low" are closer to "low" than to "high", as suggested by mouse trajectories and decoding analyses, supporting the hypothesis about the mitigation, rather than inversion, effect of negation onto the adjective. The fact that the effect of both negation and adjective processing can be tracked in almost overlapping temporal windows suggests that the two elements are processed in parallel, and that its mitigating effect is adopted

from early stages. Finally, mitigating effects are in line with suppression mechanisms usually associated with increased synchronization in beta-band activity in sensorimotor areas.
The study is methodologically sound, both its design and data analysis procedures are adequate for the research questions addressed. The results reported are robust: behavioral data concerning the detrimental effect of negation on response times are confirmed both by a replication study and by the behavioral part of the MEG data and are in line with the suppression mechanism that beta-band activity suggests. The two decoding approaches converge on one of the four hypotheses formulated (mitigation). Finally, the manuscript is clear and well organized, and I enjoyed reading it very much. So, in what follows, I can only outline some minor comments that I think can contribute to clarifying some of the tested hypotheses and the rationale of the study, as well as the discussion of the results.

We very much appreciate the reviewer's encouraging feedback.

One of the goals of the study is to test the effect of negation on lexical-semantic representations of antonyms and the time course of this effect. Nevertheless, three of the four hypotheses formulated (page 14) are not explicitly linked to any temporal course. Following psycholinguistic evidence, Hypothesis 1(H1) tests the possibility that the interpretation of negated phrases follows a two-step process, whereby at initial stages of interpretation, negation does not affect the meaning of the adjective (not good = good), but it does so at later stages. What about H2, H3 and H4?

Thank you for raising this good point. The hypotheses that we formulated in Fig.6A derive from previous linguistic and psycholinguistic accounts (including the two-steps account), however it is true that they do not make specific assumptions about time, but rather focus on *how* negation affects meaning. The temporal unfolding of the effect of negation can be derived as a combination of different hypotheses, in the case of the two-steps account, by combining first *no effect* and then *inversion* (or mitigation): at first "not good" = "good" and later "not good" = "bad". Overall, our study thus addresses this gap-of-knowledge, and starts to provide a precise temporal unfolding of this kind of minimal composition.

We have added a paragraph to the Results and Discussion that further clarifies this point:

"Importantly, these predictions focus on how negation affects representations rather than on when. Thus, a combination of mechanisms may be observed over time (e.g., first no effect and then inversion)." [lines 437-439]

"These four hypotheses make predictions about how negation operates on scalar adjectives at any given time. It is thus possible that multiple mechanisms may unfold over time when looking at time-resolved data (e.g., first no effect and then inversion)." [lines 589-592]

When is mitigation expected to occur? From early stages (countering H1) or at later stages (in line with H1)?

We thank the reviewer for the opportunity to clarify this point. Our approach consisted of illustrating predictions about how negation operates on scalar adjectives and evaluating the time unfolding based on our results. There exists contrasting evidence about the temporal dynamics of the effect of negation, either showing that negation operates in two-steps or in one incremental step. Our neural data point to effects of negation at early lexico-semantic processing stages, thus they do not support a two-step account wherein the initial representation of negated adjectives would not be affected by negation. In other words, our results do not support H1 (i.e., “no effect”) at early stages. While our data support the hypothesis that, broadly, negation mitigates the representation of adjectives at early processing stages, our design does not allow us to pinpoint to the specific lexico-semantic features upon which negation operates. We have now clarified this point in the Discussion and expanded on the effect of negation over time:

“This result rules out prediction (1) No effect and supports prediction (2) Mitigation, wherein negation weakens the representation of adjectives. We observed such a reduction in early representations (i.e., within ~300 ms from adjective onset). This finding is consistent with previous research that reported effects of negation as soon as lexical-semantic representations of words are formed [12,29–31,71], and not exclusively at later processing stages (e.g., P600 [72,73]). In addition, the fact that low vs. high adjectives are decodable ~400 ms after the adjective onset in negated phrases (Fig.6C, Fig.S4A, Fig.S4B) raises two novel questions: First, is the mitigation effect of negation stable over time? Second, at what exact stages does it operate upon (see [64,66])? Using a masked priming paradigm, van Gaal et al. [33] analyzed participants' EEG responses to sequences of words that were either consciously or unconsciously perceived. Their findings indicate that the meaning of multiple words, including negation, can be integrated even when subjects report not seeing them, but that conscious perception is required for later grammatical integration. Future research remains necessary to more precisely tease apart the lexical, semantic, and syntactic features that are selectively affected by the negation operator over time.
Taken together, our behavioral and neural data jointly point to a mitigation rather than an inversion effect of negation at early semantic processing stages, and exclude the hypothesis according to which negation does not change the representation of antonyms. Specifically, these results show that initial interpretations and early neural representations of negated adjectives are similar to that of affirmative adjectives, but weakened.” [lines 598-617]

Similarly, are inversion and change expected to occur early or late? The temporal course of negation effect is not made explicit either when introducing the results or when describing the predictions following the use of the two decoding approaches (page 29). Relatedly, overall the results clearly point to the mitigating effect of negation to be occurring in parallel to adjective processing, thus countering two-steps mechanisms. Yet, this aspect is not explicitly tackled in the introduction, and I think it is worth highlighting.

We thank the reviewer for raising this important point. We have expanded the Introduction to address this point:

"The combined behavioral and neurophysiological data adjudicate among these hypotheses and identify potential mechanisms that underlie how negation functions in online meaning construction. Emerging temporal dynamics clarify how the effect of negation on adjective meaning unfolds over time, whether incrementally (i.e., in parallel to adjective processing) or serially (i.e., in a second step after adjective processing)." [lines 129-133]

Very minor comments:

- A second comment concerns the set of hypotheses that the two decoding analyses can test. For decoding approach 1, there does not seem to be any clear boundary between "no effect" and "mitigation" in terms of probability estimates, as illustrated in Figure 5 and stated on page 29 (lines 799-801). So this decoding approach does not clearly distinguish between H1 and H2, but it can tease them apart from H3 and H4 (and can tease these two apart as well). In contrast, decoding method 2 seems to be adopted to explicitly tease H1 and H2 apart, but it cannot discriminate between H3 and H4. So it seems to me the two approaches are adopted because they complement each other, and if this is so, this could be briefly clarified in the manuscript.

Thank you. This is exactly the case. We have clarified this in the Results section and Discussion:

"To adjudicate between these four hypotheses, we performed two complementary sets of decoding analyses." [lines 440-441]

"Decoding approach (ii) allows for a direct comparison between AUC and probability estimates in affirmative and negated phrases and to disentangle predictions (1) No effect from (2) Mitigation." [lines 448-450]

"Using two complementary decoding approaches, [...]" [line 592]

- Is there a reason why the modifier "really" was used and not "very"? My intuition is that it creates a better contrast with "not" and so a clearer polarization between two abstract meaning representations, but I would like to know it from the authors.

Thank you. We have clarified this in the Methods section:

""really" was preferred to "very" as it more strongly intensifies the meaning of the adjective, e.g., "really hot" > "very hot"" [lines 723-724]

- Line 213: "even in the absence of feedback". The description of the behavioral experiment and its results up to this point do not mention the presence of any feedback in the study. One has to read the methods at the end of the manuscript to know about it.

Thank you for catching this. We have clarified this earlier in text:

"Feedback was provided at the end of each trial (to which 1 and 0 were assigned to compute the average feedback score)." [Figure 1, legend and lines 168-169].

Furthermore, we have now added Fig.3 in the main text, which should clarify the difference between the two behavioral experiments.

- Figure 1: IWI/ITI, abbreviations are not spelled out.

Thank you, we have spelled this out in the figure legend: IWI = inter-word-interval

- Page 13, line 335: "demonstrates" should probably be "demonstrate".

Thank you.
