# Supplementary material for: Negation mitigates rather than inverts the neural representations of adjectives
Source: PLoS Biol. 2024 May 30;22(5):e3002622. doi: 10.1371/journal.pbio.3002622 (PMC11139306; doi:10.1371/journal.pbio.3002622)
Supplement: Attachment — Submitted filename: Response to Reviewers.pdf [file pbio.3002622.s011.pdf]

Response to Reviewers

We would like to thank the Reviewers and the Academic Editor for their helpful feedback and suggestions.

We have addressed the comments of Reviewer #1, discussed the limitations of the study design and interpretation, and deepened the discussion of the theoretical insights offered by our study in the Discussion. In the following, we present point-by-point responses, each followed by the changes/additions and the corresponding page numbers in the manuscript. In addition, we have highlighted all changes in orange in the manuscript.

(1) Reviewer 1's remaining points:

REVIEWS:

Reviewer #1: “I thank the reviewers for their elaborate responses. Many of my points have been addressed. I do still have a few remaining point that need addressing.”

1) The authors try to link the MEG and the behavioral data. Indeed, I agree that at the early stages of the behavioral choices the participants for the negated options divert first to the other response option (which could be indicative of mitigation). However, to better link the behavioral data to the MEG data, it would be good to show that at the decision stage there is an inversion in the MEG responses as well. This can be done by locking the data to the probe or to the response and repeating the decoding analysis. Especially considering that they have reverted the response options there is no confound of button presses here.

We thank the reviewer for this observation, and we agree that showing a later inversion in the MEG response would better support our behavioral findings. Following the reviewer’s suggestion, we have now performed the analysis presented in **Fig.6C** and locking the response to the probe and to the response time, and no significant above or below chance AUC was observed. Decoding accuracies are shown below:

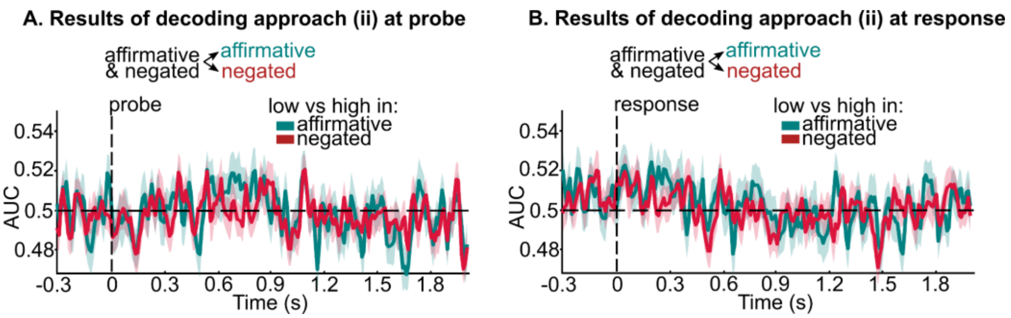

As we did not introduce this task to measure subtle changes in adjective representation as a function of negation after the probe was presented - but rather to keep participants engaged - we are not confident that we could observe changes in adjective representation at these stages. We

ran a similar analysis in **Supplementary Figure S3**. We have now acknowledged the limitations of our design more in depth in the Discussion:

*"Differences between our behavioral and neural results could be ascribed to the fact that the behavioral task had to be adapted to the MEG environment. In the behavioral experiment (and its replication), participants were continuously and explicitly indicating their interpretations in a bi-dimensional space (i.e., the semantic scale defined by the polar adjectives). Conversely, the MEG experiment allowed us to measure neural correlates of semantic representation over time (not affected by motor activity) but not the explicit interpretations, which were collected only after the probe was presented (1850 ms after the adjective presentation) and only through a yes/no decision task (**Fig.2B** and **Fig.4A**). Thus, both our designs present advantages and limitations and together offer a more encompassing approach: bi-dimensional semantic scales in the behavioral task could have masked nuanced changes in semantic representation over time, which we were able to better evaluate in the MEG data; in turn, later effects of negation might have been masked by the task employed in the MEG experiment; however, they were more carefully assessed in our behavioral approach. Design limitations are crucial when interpreting behavioral and neuroimaging findings. Adopting a multi-modality approach (e.g., behavioral mouse tracking and MEG recordings) helps overcome some of the limitations related to individual experimental approaches; our complementary approaches allowed us to advance a well-motivated interpretation." [lines 627-642]*

2) I do not feel that the authors acknowledge the problem of visual similarity ending up in the decoding sufficiently. While I acknowledge that their blocked design makes it difficult to do this analysis, I still hold that if visual features influence the decoding, it is difficult to differentiate the outcome expectations for the different models. For example, see figure 6. Let us say that the true underlying representation is a changed representation (4), but part of the decoding is due to visual differences. That would effectively increase the overall decoding (as the sensory representations are the same). This increase would lead exactly to a pattern consistent with mitigation. I do not think they can currently differentiate these options.

Thank you, we appreciate this point. We now mention this in the updated limitation paragraph in the Discussion:

*"One possible limitation is that visual features might still be deciphered and consequently affect the analyses presented in **Fig.6**. However, two factors mitigate this potential issue: first, our stimulus set by design contains words with varying visual features, which makes it difficult for a linear decoder to identify a common visual feature to rely on; second, generalized representations shown for decoding approach (i) last up to 550 ms from adjective onset, likely reflecting lexical-semantic rather than purely perceptual processing [64–68]." [lines 618-623]*

Reviewer #3: "I read the revised manuscript and the authors' rebuttal to my comments, which were satisfactorily addressed. I have no further comments and so endorse this manuscript for publication."

Thank you.

(2) Limitations of the study design and interpretation of the results more clearly

Thank you, we have now expanded on the limitations of our design and interpretation in the revised Discussion:

"Specifically, these results show that initial interpretations and early neural representations of negated adjectives are similar to that of affirmative adjectives, but weakened. One possible limitation is that visual features might still be deciphered and consequently affect the analyses presented in ***Fig.6.*** *However, two factors mitigate this potential issue: first, our stimulus set by design contains words with varying visual features, which makes it difficult for a linear decoder to identify a common visual feature to rely on; second, generalized representations shown for decoding approach (i) last up to 550 ms from adjective onset, likely reflecting lexical-semantic rather than purely perceptual processing [64–68]. The comparison between MEG and behavioral results also reveals interesting differences. Behavioral data reveal that, in negated phrases, participants later modify their initial interpretation towards, but never exactly as, the opposite meaning. Our MEG data do not show an inversion of adjective representation as a function of negation, at early or later lexical-semantic processing stages. Differences between our behavioral and neural results could be ascribed to the fact that the behavioral task had to be adapted to the MEG environment. In the behavioral experiment (and its replication), participants were continuously and explicitly indicating their interpretations in a bi-dimensional space (i.e., the semantic scale defined by the polar adjectives). Conversely, the MEG experiment allowed us to measure neural correlates of semantic representation over time (not affected by motor activity) but not the explicit interpretations, which were collected only after the probe was presented (1850 ms after the adjective presentation) and only through a yes/no decision task (****Fig.2B*** *and* ***Fig.4A****). Thus, both our designs present advantages and limitations and together offer a more encompassing approach: bi-dimensional semantic scales in the behavioral task could have masked nuanced changes in semantic representation over time, which we were able to better evaluate in the MEG data; in turn, later effects of negation might have been masked by the task employed in the MEG experiment; however, they were more carefully assessed in our behavioral approach. Design limitations are crucial when interpreting behavioral and neuroimaging findings. Adopting a multi-modality approach (e.g., behavioral mouse tracking and MEG recordings) helps overcome some of the limitations related to individual experimental approaches; our complementary approaches allowed us to advance a well-motivated interpretation." [lines 616-643]*

(3) deepen the discussion of the theoretical insights your study can offer (as suggested by Reviewer 2 and 3 in the previous round of review)

Thank you, we have added references and a paragraph to the Discussion that details the main theoretical insights that our study offers:

"Taken together, our behavioral and neural data jointly point to a mitigation rather than an inversion effect of negation at early semantic processing stages, and exclude the hypothesis according to which negation does not change the representation of antonyms (i.e., first processing step in two-step accounts [27])." [lines 613-616]

*"It is uncontroversial that negation is a fundamental operation in language and cognition. A comprehensive cognitive model of negation has been elusive. Our design (**Fig.1**), the explicit hypothesis space we consider (**Fig.6A**), and the choice of methodologies we employ derive from the careful consideration of the different theoretical frameworks [11,12,27,28,44]. To be sure, we also built on existing behavioral measures (i.e., response time and accuracies; [17]), psycholinguistic approaches (i.e., effects of negation on scalar adjectives; [42,43]), and neuroimaging domains (i.e., time, space, and frequency domains; [26,35,40]) that have been used in the study of negation. Our insights contribute decisive new data to the debate on how negation operates by showing that negation functions as a mitigator of lexical-semantic representations of scalar adjectives, possibly through general-purpose inhibitory systems. The results also speak to the question of when negation operates by showing early effects of negation on adjective processing. More broadly, we show that, by characterizing subtle changes of linguistic meaning through negation, using time-resolved behavioral and neuroimaging methods and multivariate decoding, we can tease apart different possible representational outcomes of combinatorial operations, above and beyond the sum of the processing of individual word meanings." [lines 680-694]*
